# An azobenzene container showing a definite folding – synthesis and structural investigation

**DOI:** 10.3762/bjoc.15.156

**Published:** 2019-07-10

**Authors:** Abdulselam Adam, Saber Mehrparvar, Gebhard Haberhauer

**Affiliations:** 1 Institut für Organische Chemie, Universität Duisburg-Essen, Universitätsstr. 7, D-45117 Essen, Germany

**Keywords:** azobenzene, macrocycles, molecular switch

## Abstract

The combination of photo-switchable units with macrocycles is a very interesting field in supramolecular chemistry. Here, we present the synthesis of a foldable container consisting of two different types of *Lissoclinum* macrocyclic peptides which are connected via two azobenzene units. The container is controllable by light: irradiation with UV light causes a switching process to the compact *cis,cis*-isomer, whereas by the use of visible light the stretched *trans,trans*-isomer is formed. By means of quantum chemical calculations and CD spectroscopy we could show that the *trans*→*cis* isomerization is spatially directed; that means that one of the two different macrocycles performs a definite clockwise rotation to the other, caused by irradiation with UV light. For the *cis*→*trans* isomerization counterclockwise rotations are found. Furthermore, quantum chemical calculations reveal that the energy of the *cis,cis-*isomer is only slightly higher than the energy of the *cis,trans*-isomer. This effect can be explained by the high dispersion energy in the compact *cis,cis*-isomer.

## Introduction

**Figure 1:**
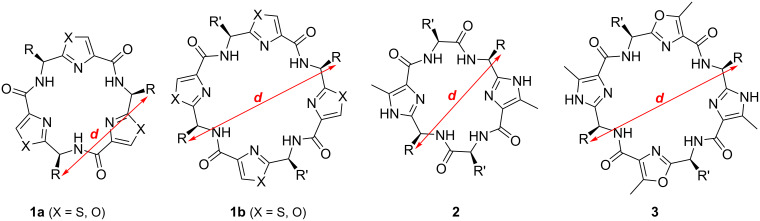
Some examples for artificial *C*_2_- and *C*_3_-symmetric platforms based on *Lissoclinum* cyclopeptide alkaloids.

In supramolecular chemistry rigid scaffolds are required to arrange different recognition units in predefined distances and spatial orientation to each other [1]. One example for such rigid systems are macrocycles which stem from *Lissoclinum* cyclopeptide alkaloids (Figure 1) [2–3]. Here, the required recognition units can be introduced via the amino acid side chains or via the side chains of the azole rings. The orientation and the distance between the recognition units are determined by the type and size of the macrocyclic platform, e.g., if all of the amino acid side chains are of the same configuration, they are presented on one face of the macrocycle in a convergent manner. The artificial *Lissoclinum* cyclopeptide platforms feature *C*_2_, *C*_3_ and *C*_4_ symmetry [3]. So far, a series of receptors based on these macrocycles were synthesized. They have been designed for the selective recognition of sulfate ions [4–6], di- and triphosphate ions [7], for sensing of pyrophosphate ions in aqueous solutions [8], as receptors for phenols [9], α-chiral primary organoammonium ions [10], and biomolecules [11–17]. Furthermore, modified *Lissoclinum* cyclopeptides were used for the construction of novel tubular and cage structures [18–19], as prototypes for mimicking multiple loops of proteins [20] and for homochiral supramolecular polymerization [21–22]. Beside the usage of the side chains of the amino acids and the azole rings for molecular recognition, the functional groups of the scaffolds of these cyclopeptides have also been applied as receptors for Y-shaped anions [23] and as ligands for copper(II) complexes [24–25].

Of special interest is the design of artificial *Lissoclinum* cyclopeptides which can be switched by the incorporation of a suitable switching unit into the scaffold. A switching process would allow to vary the orientation and the distance between the recognition units. Examples for such switching units are photochromic molecules which can be reversibly changed between two isomers of different structures [26–29]. One prominent switching unit is azobenzene and its derivatives [30–41]. The *trans*-isomer features a stretched and the *cis*-isomer has a compact geometry. In general, the *trans*→*cis* isomerization is triggered by UV light whereas the *cis*→*trans* back relaxation takes place by visible light or heat [30,42]. Due to the high reversibility, the simple synthesis and the high photostability azobenzene derivatives are the most commonly used switching units. A further advantage of the use of azobenzene as switching unit is the fact that it is possible to control the conformation of the *cis* or the *trans-*isomers by chiral bridges [43–48].

**Figure 2:**
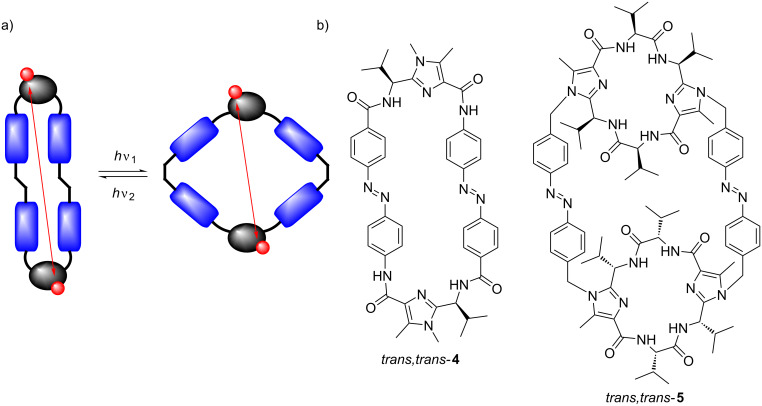
a) Principle of a chiral foldable platform and container based on *Lissoclinum* cyclopeptide alkaloids. b) Switchable molecular platform **4** and switchable molecular container **5**.

Up to now two artificial *Lissoclinum* cyclopeptides, which feature an azobenzene moiety to change the distance between the amino acid side chains, are described in the literature [49–50]. One example is the platform **4**, which consists of two imidazole building blocks connected by two azobenzene units (Figure 2) [50]. Irradiation of the platform **4** with UV light results in a *trans*→*cis* isomerization accompanied by a reduction of the distance between the two isopropyl groups. As further example the chiral foldable container **5** should be mentioned [49]. Here, two imidazole-containing macrocycles are linked to each other by two azobenzene units (Figure 2). Irradiation with UV light causes two consecutive *trans*→*cis* isomerization’s resulting in a stepwise decrease of the distance of the two macrocycles. Accordingly, the distance between the recognition units at the upper and the lower macrocycle decreases stepwise as well.

In the chiral container **5** two identical macrocycles are connected to each other. A further development would be a foldable container featuring two different macrocycles which allows to distinguish between the upper and the lower part of the container. Here, we present the synthesis and the structural investigation of a switchable chiral container in which two different *C*_2_-symmetric artificial *Lissoclinum* cyclopeptides are connected by two azobenzene bridges. A light-induced switching process leads to a spatially directed collapse of the container which can be detected by an increase of the diffusion coefficient of the molecule.

## Results and Discussion

### Synthesis of the chiral foldable container

**Scheme 1:**
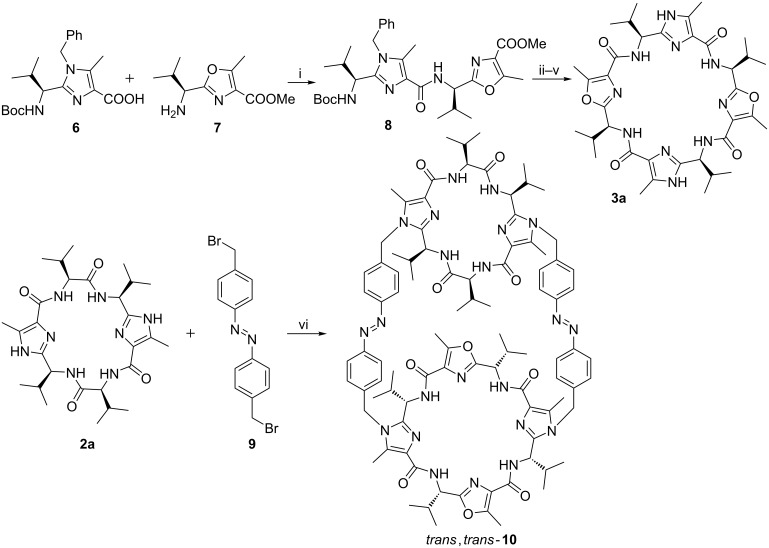
Synthesis of the chiral foldable container **10**. Reaction conditions: i) FDPP, iPr_2_NEt, CH_3_CN, 90%; ii) MeOH, dioxane, NaOH, quant.; iii) EtOAc, HCl, quant.; iv) FDPP, iPr_2_NEt, CH_3_CN, 50%; v) MeOH, Pd(OH)_2_/C, H_2_, 95%; vi) **3a**, K_2_CO_3_, CH_3_CN, Δ, 15%.

For the design of the chiral switchable container we intended to use the imidazole-containing peptides **2a** (R = R’ = iPr) and **3a** (R = R’ = iPr) as macrocycles (see Figure 1 and Scheme 1). Both feature two imidazole units which should be used to attach the azobenzene groups. Additionally, platform **2a** has two valine units, whereas platform **3a** possesses two oxazole rings. Overall, both macrocycles feature four amino acid side chains (isopropyl groups), whereby all of them are of the same configuration (*S*). Therefore, they are presented on one face of the macrocycle in a convergent manner.

Platform **3a** was synthesized in a few steps according to a known procedure (Scheme 1) [51]. Therefore, the imidazole-containing acid **6** was reacted with the free amine **7** using pentafluorophenyl diphenylphosphinate (FDPP) as coupling reagent resulting in the formation of amide **8**. After saponification of the methyl ester and removal of the Boc protective group, the resulting amino acid was cyclodimerized to the benzyl-protected macrocycle. The yield for the cyclization amounts to about 50%. The last step was the removal of the benzyl group by hydrogenolysis to yield the desired macrocycle **3a**. The cyclopeptide **2a** [52] is commercially available.

Initially, we tried to synthesize the chiral foldable container **10** starting from the macrocycles **2a** and **3a** in a stepwise manner. That means, we intended to react in a first step one macrocycle with two azobenzene units having each one reactive and one protected group. In a second step we wanted to transform the protected groups at the azobenzene units into reactive groups. The latter should react in a third step with the other macrocycle. However, although we varied the reaction sequence regarding the used macrocycles, none of these reaction pathways led to the desired molecule. Therefore, we changed our strategy and we tried to synthesize the chiral foldable container **10** in a one pot reaction. For this purpose, the platforms **2a** and **3a** and the dibromide **9** were dissolved in acetonitrile in the ratio 1:1:2.2. To this solution potassium carbonate as base was added and the whole mixture was refluxed for one day. Fortunately, the desired container was formed in a yield of 15%, which is astonishingly good considering the multiple reaction paths. As byproducts the containers consisting of each two identical macrocycles **2a** and **3a**, respectively, are formed. These containers could not be separated from each other. The isolation of the desired container was achieved by column chromatography followed by HPLC. It is noteworthy that the synthesis of this container showing two different *Lissoclinum* cyclopeptides only took a couple of steps starting from an imidazole and an oxazole building block, an azobenzene unit as well as commercially available compounds.

### Investigation of the structure and the switching process

**Figure 3:**
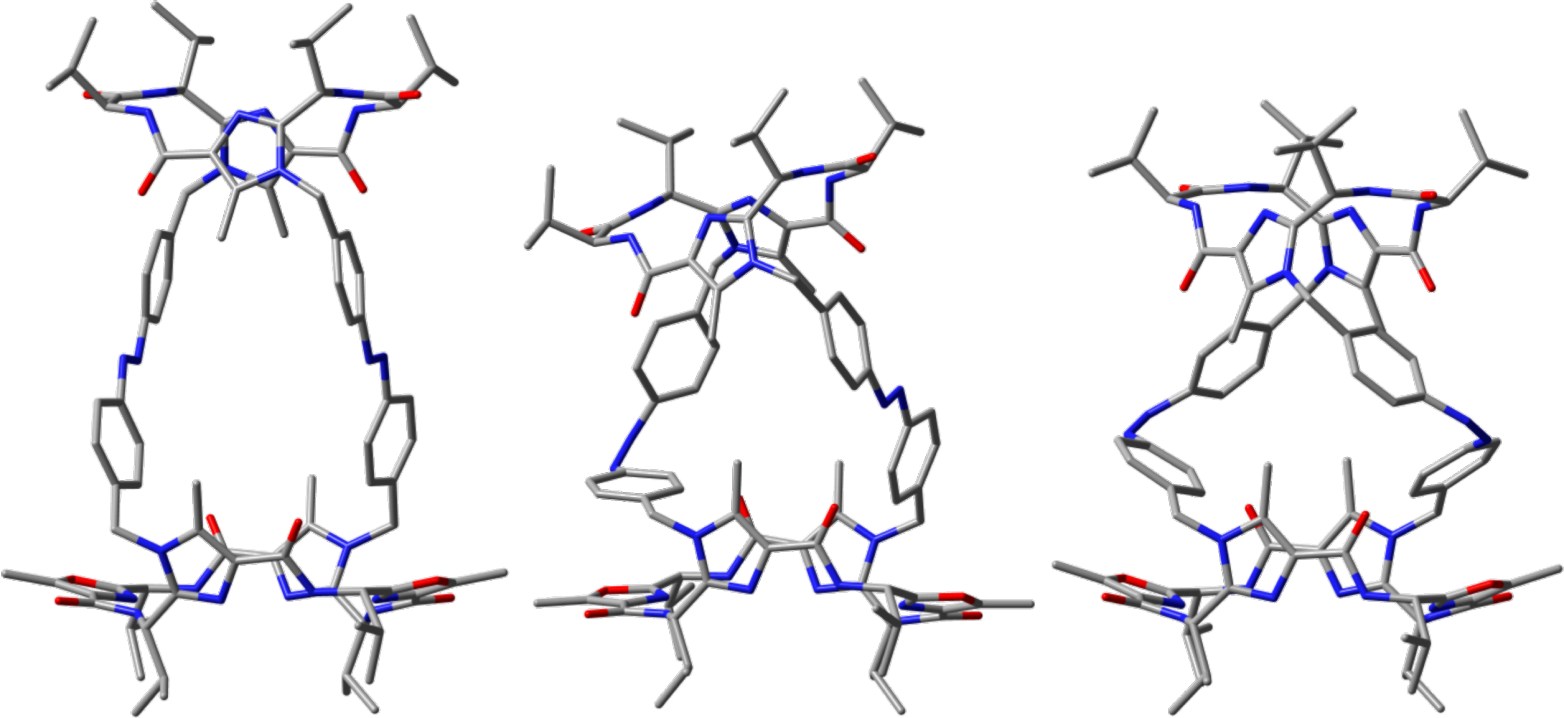
Molecular structures of *trans*,*trans*-**10** (left), *cis*,*trans*-**10** (middle) and *cis*,*cis*-**10** (right) calculated by means of B3LYP/6-31G*. All hydrogen atoms are omitted for the sake of clarity.

To investigate the structures of the foldable container **10** in the gas phase, the geometric parameters of the *trans*,*trans-*, *cis*,*trans-* and *cis*,*cis*-isomers were fully optimized by means of the DFT potentials B3LYP [53–55] and B3LYP-D3 [56–57]. The latter includes an additional dispersion correction and describes dispersion interactions more accurately for larger atomic distances. As basis set 6-31G* [58–59] was applied. In the case of the *cis*,*trans-* and *cis*,*cis*-isomers we tried to calculate all possible conformations [*cis*,*trans*-(*M*), *cis*,*trans*-(*P*), *cis*,*cis*-(*M*,*M*), *cis*,*cis*-(*M*,*P*) and *cis*,*cis*-(*P*,*P*)]. However, it turned out that the *P* conformers represent no minima on the potential energy surface. Furthermore, single point calculations by means of the density functionals B3LYP and B3LYP-D3 were performed by using the basis set def2-TZVP [60–61]. The thus obtained data are listed in Table 1. The calculated structures are shown in Figure 3 and Figures S1 and S2 in Supporting Information File 1. For comparison, the data for azobenzene were also calculated using the same level of theory and are listed in Table 1.

**Table 1:** Relative energies [kcal/mol] of the isomers of **10** and azobenzene calculated using different methods.

compound	Δ*E*^a^	Δ*E*^b^

		
*trans*,*trans*-**10**	0.0	0.0
*cis*,*trans*-**10**	20.0	8.3
*cis*,*cis*-**10**	34.0	10.2
*trans-*azobenzene	0.0	0.0
*cis-*azobenzene	15.1	12.6

^a^B3LYP/def2-TZVP//B3LYP/6-31G*. ^b^B3LYP-D3/def2-TZVP//B3LYP-D3/6-31G*.

A comparison of the data for azobenzene and the chiral container **10** calculated by means of B3LYP/def2-TZVP shows that the *trans*→*cis* isomerization of *trans*,*trans*-**10** is associated with an energy increase of 20 kcal/mol (Table 1). The energy difference between *trans-*azobenzene and *cis-*azobenzene calculated at the same level of theory amounts to only 15.1 kcal/mol. That means that the *trans*→*cis* isomerization of *trans*,*trans*-**10** is accompanied by an introduction of additional strain energy of about 5 kcal/mol. For the transition from *trans*,*trans*-**10** to *cis*,*cis*-**10** an energy of 34.0 kcal/mol is required. This is ca. 4 kcal/mol more than twice the energy of the *trans*→*cis* isomerization of azobenzene. Accordingly, the *cis*,*cis*-**10** exhibits an additional strain energy of about 4 kcal/mol compared to *trans*,*trans*-**10**.

A completely different picture emerges when the dispersion correction D3 is taken into account (B3LYP-D3/def2-TZVP; Table 1). The *trans*→*cis* isomerization of *trans*,*trans*-**10** to *cis*,*trans*-**10** is energetically favored (8.3 kcal/mol) compared to the transition from *trans-*azobenzene to *cis-*azobenzene (12.6 kcal/mol). The energy input for the switching process between *cis*,*trans*-**10** to *cis*,*cis*-**10** amounts to only 1.9 kcal/mol. The reason for that is the high gain of attractive dispersion interactions due to the compact structure of the *cis*,*cis*-isomer. Therefore, we expected that the switching process from *cis*,*trans*-**10** to *cis*,*cis*-**10** is more easily realizable by an extern light stimulus than the transition from *trans*,*trans*-**10** to *cis*,*trans*-**10**. This would be very much in line with our idea to design an chiral container which can be switched between two main states (*trans*,*trans* and *cis*,*cis*).

The structures of the three isomers of container **10** calculated by means of B3LYP/6-31G* are depicted in Figure 3. As expected, the connection of the two macrocycles **2a** and **3a** via two *trans*-azobenzenes (*trans*,*trans*-**10**) results in a longer distance between the macrocycles compared to a connection via two *cis*-azobenzenes (*cis*,*cis*-**10**). The distance between the centers of the two macrocycles in the *trans*,*trans*-isomer amounts to 14.1 Å. For *cis*,*cis*-**10** a decrease of this distance to 11.3 Å is calculated. The corresponding value for the *cis*,*trans* isomer amounts to 11.8 Å, which is only slightly larger compared to the data of *cis*,*cis*-**10**. Therefore, according to the calculations the chiral container **10** should show the desired change caused by extern stimulation.

It is notable that the switching process from *trans*,*trans*-**10** to *cis*,*cis*-**10** is accompanied by a clockwise rotation of the two macrocycles towards each other. This could be explained as follows: In *trans*,*trans*-**10** the two azobenzene bridges are not perpendicularly arranged to the macrocycles, but they show a left-hand twist (Figure 3 and Figure S1 in Supporting Information File 1). The *trans*→*cis* isomerization enhances this left-hand twist leading to a clockwise rotation of the two macrocycles towards each other.

**Figure 4:**
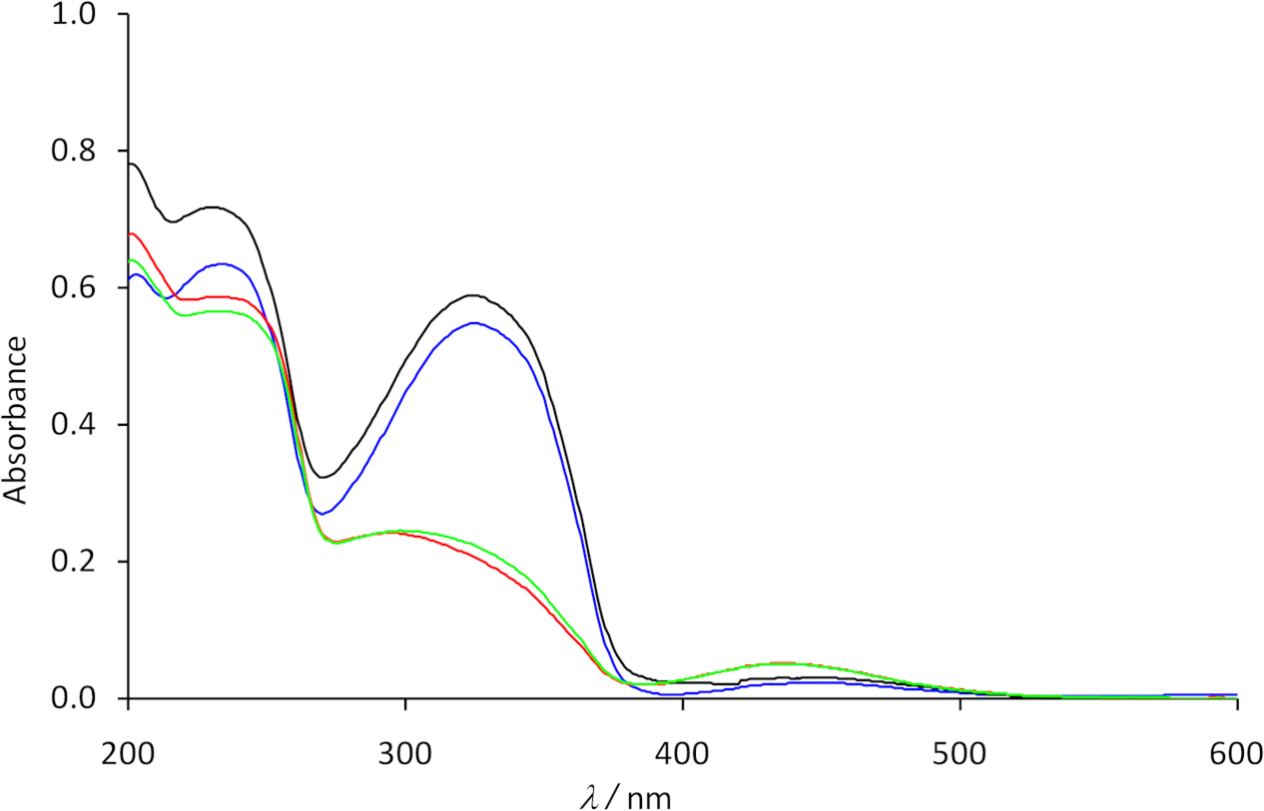
UV spectra of the foldable container **10** in acetonitrile after synthesis (blue), after irradiation with light of the wavelength λ = 365 nm (red), after irradiation with light of the wavelength λ = 405 nm (black) and after irradiation with light of the wavelength λ = 365 nm (green).

To prove the switching process experimentally, the UV spectra of the container **10** in acetonitrile as solvent were recorded (Figure 4). After the synthesis, the UV spectrum of the container shows an intensive band at 323 nm and a weak band at ca. 450 nm. The absorption at 323 nm corresponds to the π→π* transition, the second one is caused by the n→π* transition. Irradiation of the solution with UV light of the length λ = 365 nm leads to a strong decrease of the absorption band at 323 nm and to a hypsochromic shift of the n→π* transition band to ca. 430 nm (Figure 4). These changes are typical for the transition of *trans*-azobenzene units to the corresponding *cis*-isomers. A back-isomerization could be achieved by irradiation of the solution with light of the wavelength λ = 405 nm. The switching process could be repeated several times without a significant change of the spectra.

**Figure 5:**
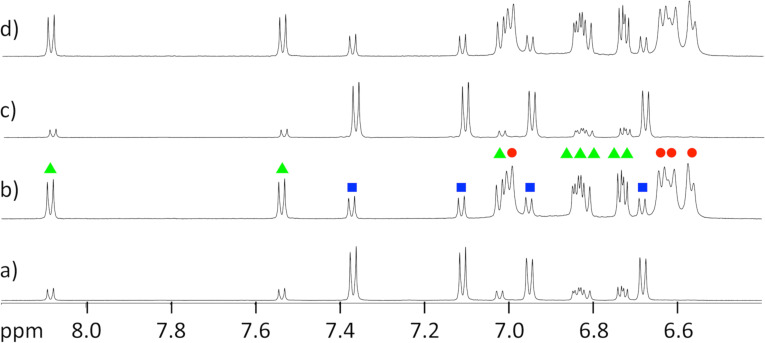
Section from the ^1^H NMR spectra of the foldable container **10** in MeOD at 600 MHz: a) after synthesis, b) after irradiation with light of the wavelength λ = 365 nm, c) after irradiation with light of the wavelength λ = 405 nm and d) after irradiation with light of the wavelength λ = 365 nm. The protons of the isomers *trans*,*trans*-**10** (blue squares), *cis*,*trans*-**10** (green triangles) and *cis*,*cis*-**10** (red circles) are marked.

In order to determine the ratio of the three isomers of the container **10** in dependence on the used light, the whole process was investigated by using HPLC and NMR spectroscopy. In both cases methanol was used as solvent. The switching processes caused by light were carried out with LED lamps and the solution was irradiated until the photostationary states were reached. In Figure 5 the ^1^H NMR spectra of the container **10** are depicted. After the synthesis, the foldable container is predominantly present as *trans*,*trans*-isomer (Figure 5a). The other signals in the spectrum stem from the *C*_1_-symmetric *cis*,*trans*-isomer, which can easily be recognized by the large number of signals. The ratio *trans*,*trans*/*cis*,*trans*/*cis*,*cis* was determined to be 68:31:<1.

Irradiation with light of the wavelength λ = 365 nm results in the formation of the *cis*,*trans-* and *cis*,*cis*-isomers. Accordingly, a *trans*,*trans*/*cis*,*trans*/*cis*,*cis* ratio of 12:39:49 can be found (Figure 5b). If this mixture is now irradiated with light of the wavelength λ = 405 nm the *trans*,*trans*-isomer is formed back and the *trans*,*trans*/*cis*,*trans*/*cis*,*cis* ratio changes to 77:22:<1 (Figure 5c). The switching process can be repeated without a significant change of the spectrum (Figure 5d; *trans*,*trans*/*cis*,*trans*/*cis*,*cis* ratio = 11:39:50).

The HPLC spectra of the investigation of the switching process are shown in the Supporting Information File 1 (Figures S3–S6). A comparison of the corresponding ^1^H NMR spectra shows that the extent of the switching process is dependent on the concentration of the azo compound. The more diluted the solution, the larger are the changes caused by the LED lamps. This effect is already known for this kind of switches [49–50]. The *trans*,*trans*/*cis*,*trans*/*cis*,*cis* ratio of the solution after synthesis amounts to 63:35:2 and resemble the ^1^H NMR data (68:31:<1). After irradiation with light of the wavelength λ = 365 nm a *trans*,*trans*/*cis*,*trans*/*cis*,*cis* ratio of 4:26:70 is observed, which is distinctly higher than that found using ^1^H NMR spectroscopy (12:39:49). Also the back-isomerization caused by light of the wavelength λ = 405 nm results in an higher amount of the *trans*,*trans*-isomer (*trans*,*trans*/*cis*,*trans*/*cis*,*cis* = 80:19:1). It is also possible to get a mixture having the *cis*,*trans*-isomer as main component, if the solution is exposed to light of the wavelength λ = 530 nm (*trans*,*trans*/*cis*,*trans*/*cis*,*cis* = 8:60:32).

**Figure 6:**
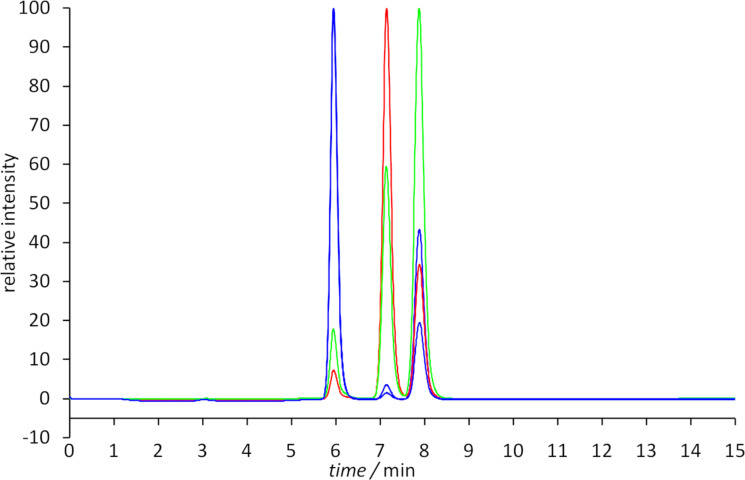
HPLC spectra (ReproSil Phenyl, 5 μm, 250 × 8 mm; methanol) of *trans*,*trans*-**10** (blue), *cis*,*trans*-**10** (green) and *cis*,*cis*-**10** (red) 20 min after isolation by means of HPLC.

The use of preparative HPLC allows the isolation of the single isomers and the investigation of the separated compounds. Therefore, the single HPLC peaks were collected and measured within a few minutes by CD spectroscopy. The purity of the separated isomers was tested as follows: after collection of the single HPLC peaks and a waiting time of about 20 min, HPLC chromatograms of the single fractions were recorded. These chromatograms show a purity of >93% for each isomer (Figure 6).

**Figure 7:**
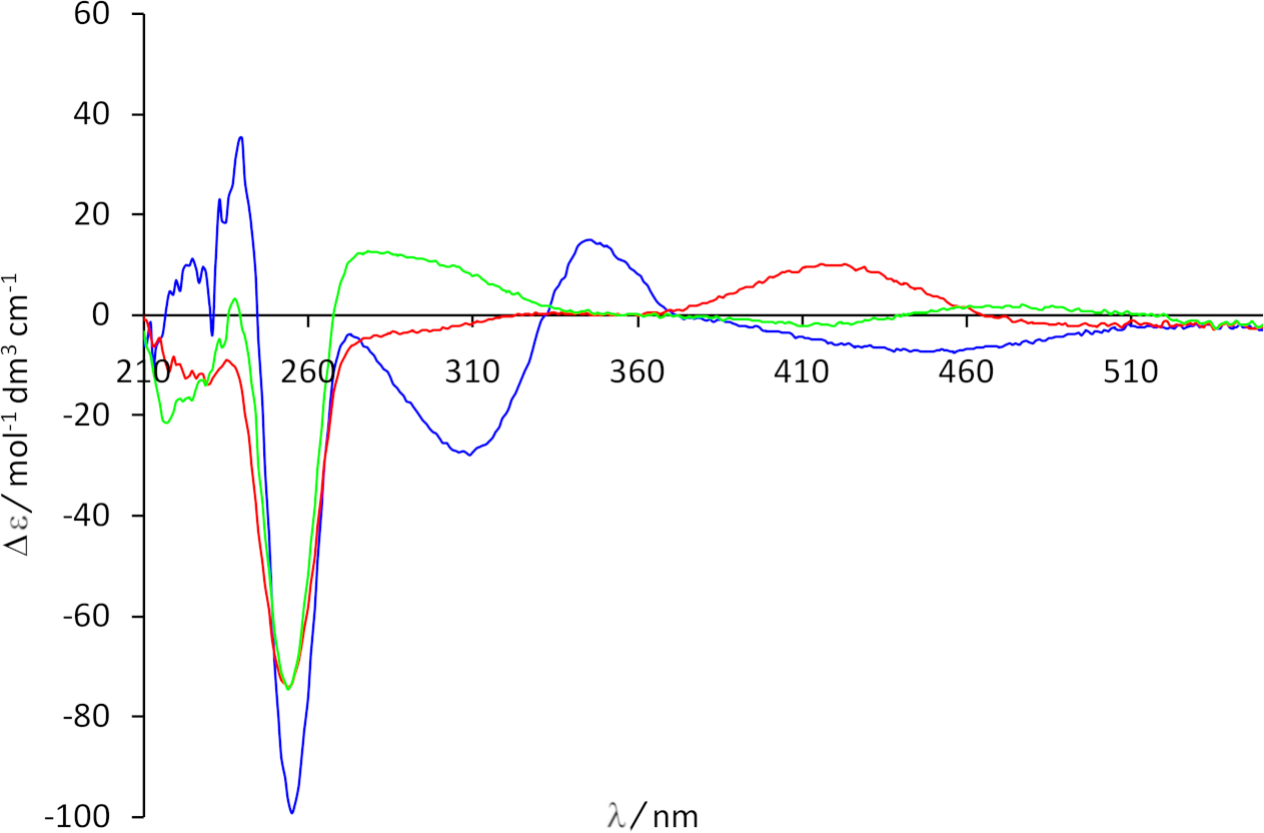
CD spectra of *trans*,*trans*-**10** (blue), *cis*,*trans*-**10** (green), and *cis*,*cis*-**10** (red) in methanol (*c* = 3.0 × 10^−5^ M).

The CD spectra of the single isomers of the container **10** are shown in Figure 7. It should be mentioned that the area around 450 nm of the CD spectra of simple alkyl-substituted *cis*-azobenzene derivatives is dominated by only one transition (n→π*). Accordingly, the conformation (*M* or *P*) of the *cis*-azobenzene moiety can directly be identified from the sign of the Cotton effect at 450 nm. Previous studies have demonstrated that the *cis*-(*M*) isomer shows a positive and the *cis*-(*P*) isomer has a negative Cotton effect in this region [43,49]. If this is taken into account, it becomes obvious that the *cis*,*cis*-isomer adopts the *M*,*M* conformation. This is in line with the DFT calculations finding only the *cis*,*cis*-(*M*,*M*) isomer as minimum on the energy potential surface. The spectrum of *cis,trans-***10** allows the conclusion that the *cis*-azobenzene unit is present in its *M* conformation. For the *P* conformation we would expect a negative Cotton in the area around 450 nm. That means the chiral information is transferred from both macrocycles to the *cis*-azobenzene units in *cis*,*trans*-(*M*)-**10** and *cis*,*cis*-(*M*,*M*)-**10** and the two different macrocycles perform a definite clockwise rotation to the other caused by irradiation with UV light and a counterclockwise rotation when the compound is exposed to visible light.

**Figure 8:**
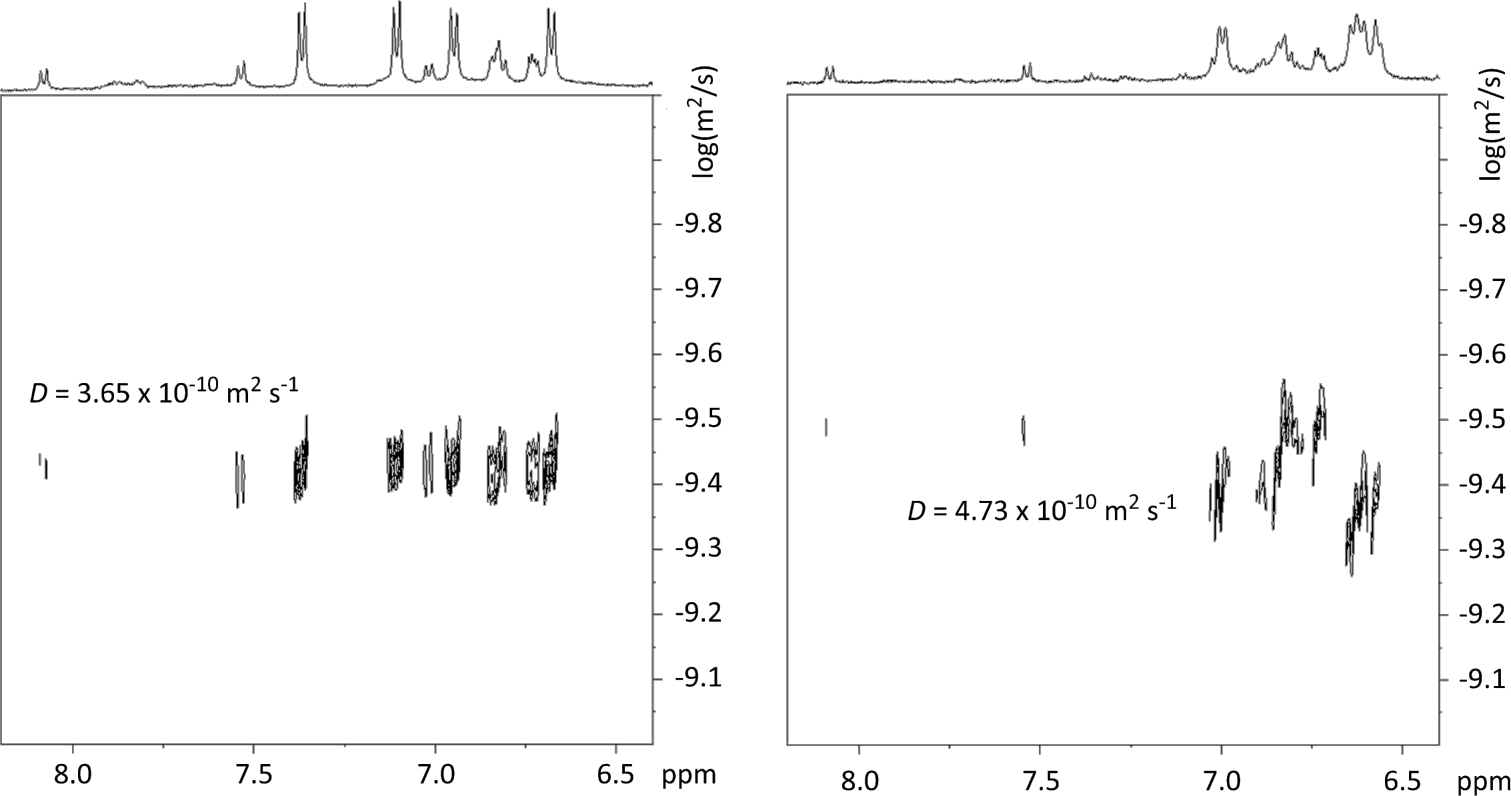
DOSY NMR spectra (500 MHz in MeOD at 25 °C) of the foldable container **10** after synthesis (left) and after irradiation with light of the wavelength λ = 365 nm (right).

The spatial change of the container **10** caused by the switching process could have an impact on the size of the diffusion coefficient of the compound. To examine this, DOSY spectra of the container **10** after synthesis and after irradiation with light of the wavelength λ = 365 nm were recorded (Figure 8). Please note, that a change in the geometry of a switch need not result in a change of the size of the diffusion coefficient. For example, neither for the switchable platform **5** nor for the foldable container **6** a significant change of the diffusion coefficients caused by the switching process could be detected in the DOSY spectra of the compounds. This can be explained as follows: The DOSY NMR experiment measures the average dimension of the structures of the isomers which, summed up over all directions, could be very comparable. However, a comparison of the DOSY spectra of *trans,trans-***10** and *cis,cis-***10** shows that the diffusion coefficient of the elongated *trans*,*trans*-**10** is indeed larger than that of the more compact *cis*,*cis*-isomer (Figure 8).

## Conclusion

In conclusion, we were able to synthesize a foldable container consisting of two different types of *Lissoclinum* macrocyclic peptides which are connected via two azobenzene units. The synthesis of this container was achieved by a one pot reaction of the two imidazole-containing macrocycles and the azobenzene bridges having two reactive bromides. The desired container could be isolated in a fair yield taking the multiple reaction pathways into account. Subsequent investigations by means of quantum chemical calculations, UV, CD and NMR spectroscopy revealed that the container can be switched using UV light from the *trans*,*trans*-isomer into the *cis*,*cis*-isomer. Irradiation with visible light results in the back-isomerization. The switching process is spatially directed, accompanied by a change in the diffusion coefficient and in the distance between the centers of the two macrocycles: In the elongated *trans*,*trans*-isomer this distance shows a value of 14.1 Å, in the more compact *cis*,*cis*-isomer the distance amounts to 11.3 Å. The replace of the isopropyl groups by recognition units and the enlargement of the two linkers, which makes a shielding of a guest from the environment possible, should lead to containers which are due to their foldable feature promising candidates for applications in supramolecular chemistry.

## Experimental

**General remarks**: All chemicals were reagent grade and were used as purchased. Reactions were monitored by TLC analysis with silica gel 60 F254 thin-layer plates. Flash chromatography was carried out on silica gel 60 (230–400 mesh). ^1^H and ^13^C NMR spectra were measured with an Avance HD 600 spectrometer. All chemical shifts (δ) are given in ppm. The spectra were referenced to the peak for the protium impurity in the deuterated solvents indicated in brackets in the analytical data. HRMS spectra were recorded with a Bruker BioTOF III Instrument. UV–vis absorption spectra were obtained with Jasco J-815 and V-550 spectrophotometers. CD absorption spectra were recorded with a Jasco J-815 spectrophotometer. The IR absorption spectrum was recorded with a Varian 3100 FTIR spectrophotometer. The macrocycle **3a** was synthesized according to a known procedure [51]. The macrocycle **2a** was purchased from Squarix GmbH.

**Chiral container *trans*,*trans*-10:** To a solution of macrocycle **3a** (128 mg, 0.178 mmol), macrocycle **2a** (99 mg, 0.178 mmol), and azobenzene **9** (144 mg, 0.391 mmol) in acetonitrile (225 mL), potassium carbonate (491 mg, 3.554 mmol) was added and the mixture was refluxed at 85 °C for 25 h under an argon atmosphere. After cooling to room temperature, the solvent was evaporated to dryness, the residue was dissolved in DCM and washed with water. The aqueous layer was saturated with NaCl and then repeatedly extracted with DCM. The organic layers were combined, dried over MgSO_4_ and concentrated in vacuo. Afterwards, the residue was purified by flash column chromatography with silica gel (DCM/EtOAc/MeOH 75:25:5) and *trans*,*trans*-**10** was obtained as an orange solid (47 mg, 28 μmol, 15%). Mp >250 °C; ^1^H NMR (600 MHz, MeOD) δ 7.38 (d, ^3^*J*_H,H_ = 8.4 Hz, 4H, C_ar_H), 7.12 (d, ^3^*J*_H,H_ = 8.4 Hz, 4H, C_ar_H), 6.96 (d, ^3^*J*_H,H_ = 8.4 Hz, 4H, C_ar_H), 6.69 (d, ^3^*J*_H,H_ = 8.4 Hz, 4H, C_ar_H), 5.72 (d, ^2^*J*_H,H_ = 16.9 Hz, 2H, CH_2_C_ar_), 5.51 (d, ^2^*J*_H,H_ = 16.6 Hz, 2H, CH_2_C_ar_), 5.24 (d, ^3^*J*_H,H_ = 9.3 Hz, 2H, NHC*H*), 5.21 (d, ^2^*J*_H,H_ = 16.9 Hz, 2H, CH_2_C_ar_), 5.06 (d, ^2^*J*_H,H_ = 16.6 Hz, 2H, CH_2_C_ar_), 5.03 (d, ^3^*J*_H,H_ = 8.0 Hz, 2H, NHC*H*), 4.81 (d, ^3^*J*_H,H_ = 8.7 Hz, 2H, NHC*H*), 4.25 (d, ^3^*J*_H,H_ = 9.6 Hz, 2H, NHC*H*), 2.61–2.53 (m, 2H, C*H*(CH_3_)_2_), 2.48 (s, 6H, C_azol_CH_3_), 2.44–2.35 (m, 4H, C*H*(CH_3_)_2_), 2.28 (s, 6H, C_azol_CH_3_), 2.27 (s, 6H, C_azol_CH_3_), 2.24–2.19 (m, 2H, C*H*(CH_3_)_2_), 1.20 (d, ^3^*J*_H,H_ = 6.7 Hz, 6H, CH(C*H*_3_)_2_), 1.16 (d, ^3^*J*_H,H_ = 6.5 Hz, 6H, CH(C*H*_3_)_2_), 1.15 (d, ^3^*J*_H,H_ = 6.5 Hz, 6H, CH(C*H*_3_)_2_), 1.10 (d, ^3^*J*_H,H_ = 6.8 Hz, 6H, CH(C*H*_3_)_2_), 1.09 (d, ^3^*J*_H,H_ = 6.7 Hz, 6H, CH(C*H*_3_)_2_), 1.01 (d, ^3^*J*_H,H_ = 6.7 Hz, 6H, CH(C*H*_3_)_2_), 0.98 (d, ^3^*J*_H,H_ = 6.7 Hz, 6H, CH(C*H*_3_)_2_), 0.93 (d, ^3^*J*_H,H_ = 6.8 Hz, 6 H, CH(C*H*_3_)_2_) ppm; ^13^C NMR (151 MHz, MeOD) δ 174.0 (q, CO), 165.8 (q, CO), 165.3 (q, CO), 164.5 (q, CO), 162.9 (q, C_ar_), 154.9 (q, C_ar_), 152.8 (q, C_ar_), 152.2 (q, C_ar_), 148.6 (q, C_ar_), 148.1 (q, C_ar_), 140.8 (q, C_ar_), 140.6 (q, C_ar_), 135.5 (q, C_ar_), 135.1 (q, C_ar_), 131.4 (q, C_ar_), 131.0 (q, C_ar_), 129.7 (q, C_ar_), 128.3 (t, C_ar_), 128.1 (t, C_ar_), 124.5 (t, C_ar_), 124.2 (t, C_ar_), 62.4 (t, CHNH), 54.6 (t, CHNH), 52.4 (t, CHNH), 51.0 (t, CHNH), 48.2 (s, *C*H_2_C_ar_), 48.1 (s, *C*H_2_C_ar_), 35.9 (t, *C*H(CH_3_)_2_), 34.7 (t, *C*H(CH_3_)_2_), 33.1 (t, *C*H(CH_3_)_2_), 31.5 (t, *C*H(CH_3_)_2_), 20.3 (p, CH(*C*H_3_)_2_), 20.0 (p, CH(*C*H_3_)_2_), 19.8 (p, CH(*C*H_3_)_2_), 19.7 (p, CH(*C*H_3_)_2_), 19.6 (p, CH(*C*H_3_)_2_), 19.5 (p, CH(*C*H_3_)_2_),19.5 (p, CH(*C*H_3_)_2_), 19.2 (p, CH(*C*H_3_)_2_), 11.6 (p, C_q_*C*H_3_), 10.9 (p, C_q_*C*H_3_), 10.6 (p, C_q_*C*H_3_) ppm; IR (ATR) ν̃: 3398, 2963, 2929, 2873, 1661, 1653, 1592, 1506, 1498, 1458, 1188, 1110, 761, 715 cm^−1^; UV–vis (CH_3_CN) λ_max_ (log ε): 202 (4.69), 229 (4.69), 328 (4.50), 445 nm (2.99); HRMS (ESI–TOF) *m*/*z*: [M + H]^+^ calcd for C_92_H_115_N_22_O_10_, 1687.9161; found, 1687.9103; [M + Na]^+^ calcd for C_92_H_114_N_22_O_10_Na, 1709.8980; found, 1709.8929.

**Calculations**. All calculations were performed by using the program package Gaussian 16 [62]. The geometries of the molecules were fully optimized in the gas phase by using the DFT potentials B3LYP [53–55] and B3LYP-D3 [56–57] as well as the 6-31G* [58–59] basis set. For all calculations, the default thresholds implemented in Gaussian 16 were used. For all stationary points, no symmetry restriction was applied. The optimized geometries of all structures were characterized as minima by subsequent frequency calculations. Furthermore, the energies of the molecules were calculated using the DFT potentials B3LYP [53–55] and B3LYP-D3 [56–57] as well as the def2-TZVP [60–61] basis set.

## Supporting Information

File 1 Molecular structures, HPLC spectra of the foldable container, cartesian coordinates and absolute energies for all calculated compounds, as well as the NMR spectra of the new chiral container.
