# Supplementary material for: An azobenzene container showing a definite folding – synthesis and structural investigation
Source: Beilstein J Org Chem. 2019 Jul 10;15:1534–44. doi: 10.3762/bjoc.15.156 (PMC6633880; doi:10.3762/bjoc.15.156)

BEILSTEIN JOURNAL OF ORGANIC CHEMISTRY

Supporting Information

for

An azobenzene container showing a definite folding – synthesis and structural investigation

Abdulselam Adam, Saber Mehrparvar and Gebhard Haberhauer

Beilstein J. Org. Chem. **2019,** *15,* 1534–1544. doi:10.3762/bjoc.15.156

Molecular structures, HPLC spectra of the foldable container, cartesian coordinates and absolute energies for all calculated compounds, as well as the NMR spectra of the new chiral container

License and Terms: This is a supporting information file under the terms of the Creative Commons Attribution License (http://creativecommons.org/licenses/by/4.0). Please note that the reuse, redistribution and reproduction in particular requires that the authors and source are credited.
The license is subject to the *Beilstein Journal of Organic Chemistry* terms and conditions: (https://www.beilstein-journals.org/bjoc)

1. **Molecular structures** **S2**
2. **HPLC spectra** **S3**
3. **Absolute energies and cartesian coordinates of all calculated compounds** **S7**
4. **^{1}H NMR and ^{13}C NMR spectra of the chiral container 10** **S32**

1. Molecular structures

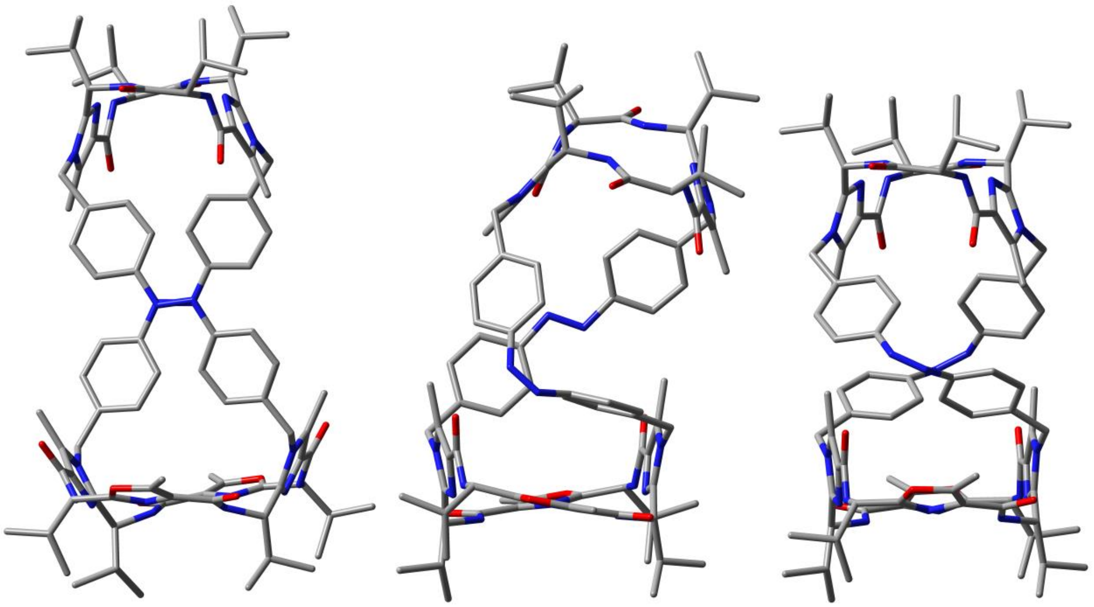

Figure S1. Molecular structures of *trans*,*trans*-**10** (left), *cis*,*trans*-**10** (middle) and *cis*,*cis*-**10** (right) calculated by means of B3LYP/6-31G*. All hydrogen atoms are omitted for the sake of clarity.

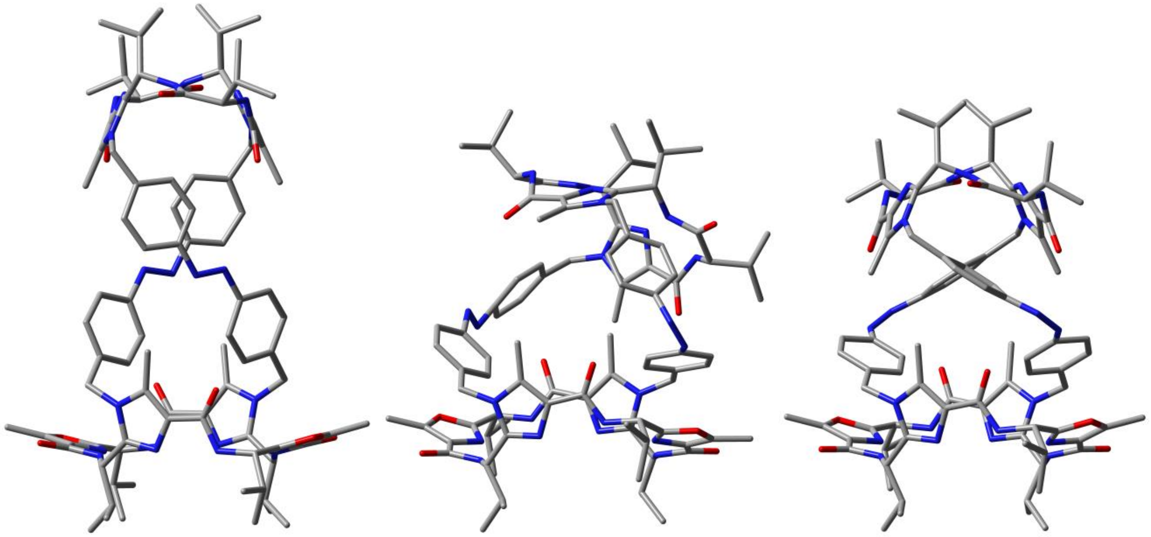

Figure S2. Molecular structures of *trans*,*trans*-**10** (left), *cis*,*trans*-**10** (middle) and *cis*,*cis*-**10** (right) calculated by means of B3LYP-D3/6-31G*. All hydrogen atoms are omitted for the sake of clarity.

2. HPLC spectra

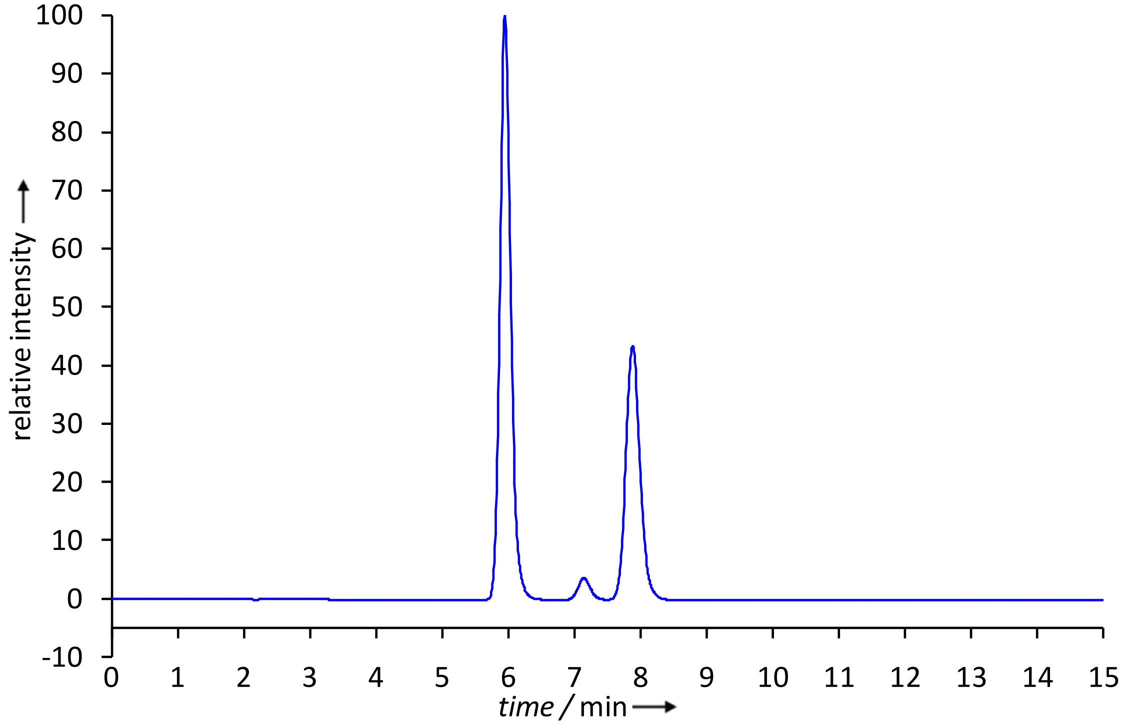

Figure S3. HPLC spectrum (ReproSil Phenyl, 5 µm, 250 × 8 mm; methanol) of the chiral container **10** after synthesis. The ratio between *trans*,*trans*-**10**, *cis*,*trans*-**10** and *cis*,*cis*-**10** amounts to 63:35:2.

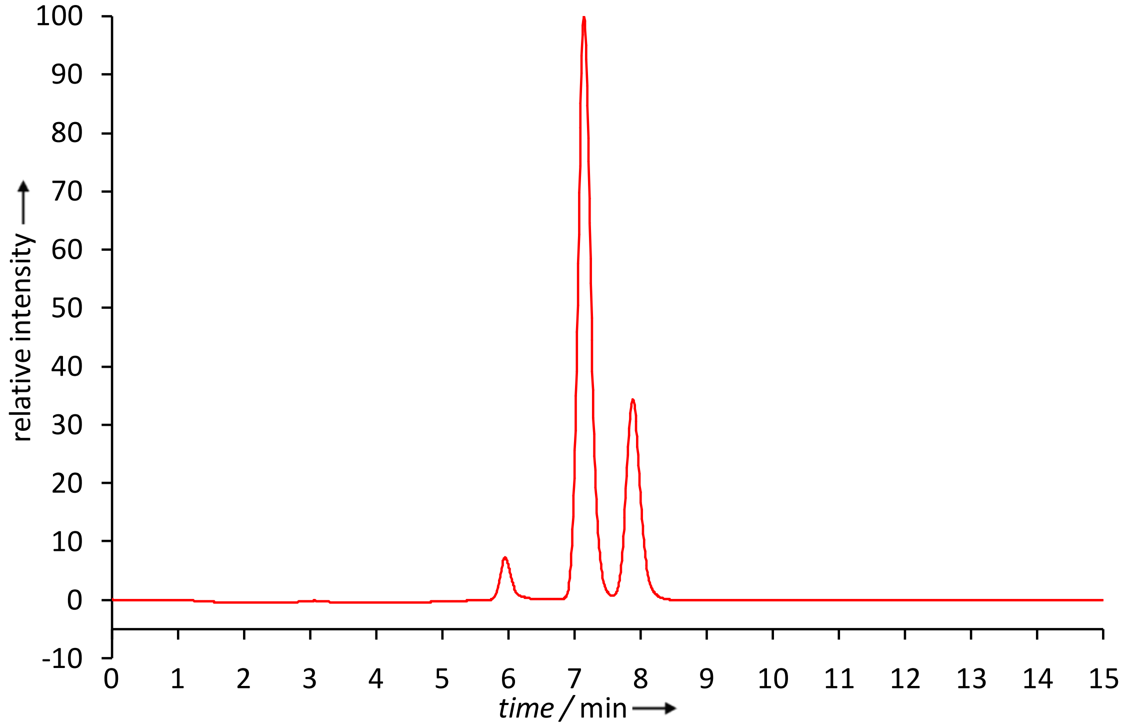

Figure S4. HPLC spectrum (ReproSil Phenyl, 5 µm, 250 × 8 mm; methanol) of the chiral container **10** after irradiation with UV light (λ = 365 nm). The ratio between *trans*,*trans*-**10**, *cis*,*trans*-**10** and *cis*,*cis*-**10** amounts to 4:26:70.

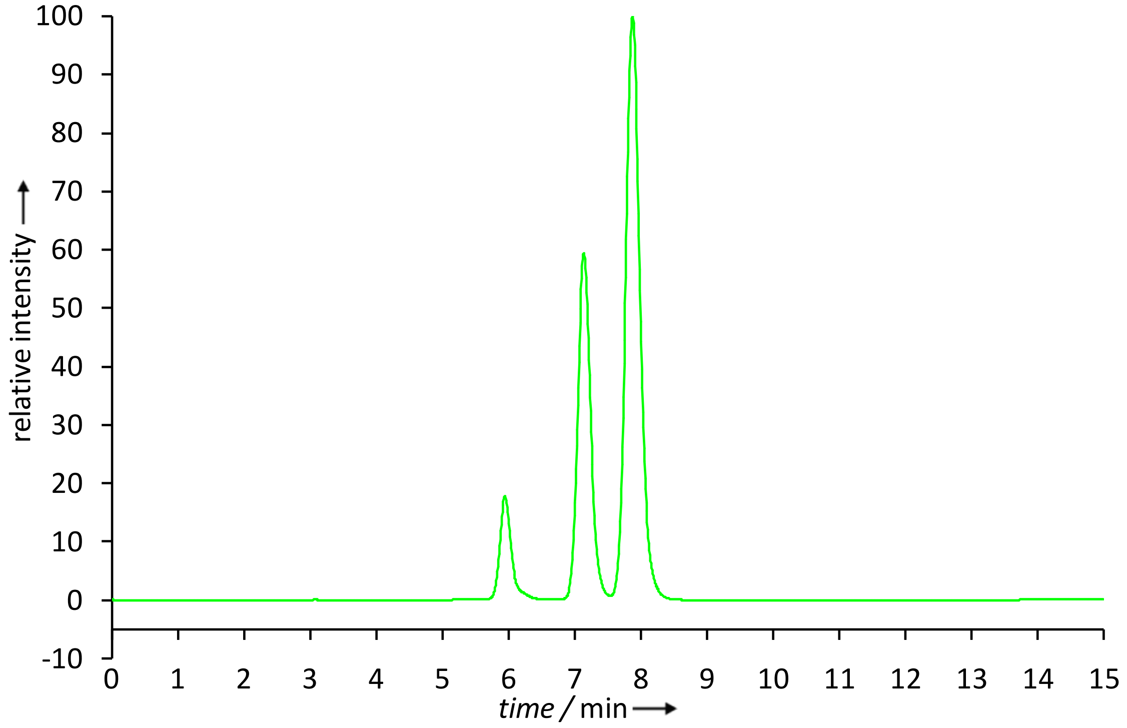

Figure S5. HPLC spectrum (ReproSil Phenyl, 5 µm, 250 × 8 mm; methanol) of the chiral container **10** after irradiation with UV light (λ = 530 nm). The ratio between *trans*,*trans*-**10**, *cis*,*trans*-**10** and *cis*,*cis*-**10** amounts to 8:60:32.

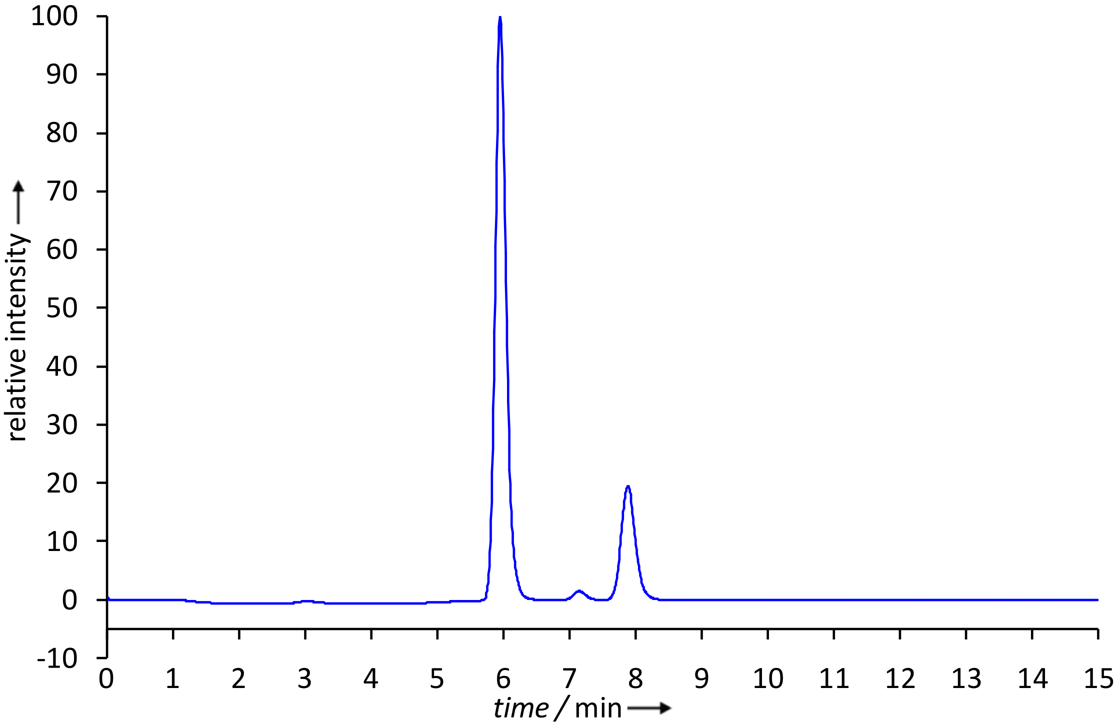

Figure S6. HPLC spectrum (ReproSil Phenyl, 5 µm, 250 × 8 mm; methanol) of the chiral container **10** after irradiation with UV light (λ = 405 nm). The ratio between *trans*,*trans*-**10**, *cis*,*trans*-**10** and *cis*,*cis*-**10** amounts to 80:19:1.

3. Absolute energies and cartesian coordinates of all calculated compounds

Table S1. Absolute energies [au] of all calculated compounds using different methods.

Molecule	E^a	E^b
trans,*trans*-**10**	-5531.468488	-5533.515093
cis,*trans*-**10**	-5531.440926	-5533.483300
cis,*cis*-**10**	-5531.419842	-5533.460916
trans-azobenzene	-572.762004	-572.971865
cis-azobenzene	-572.737793	-572.947790

[a] B3LYP/6-31G*. [b] B3LYP/def2-TZVP//B3LYP/6-31G*.

Table S2. Absolute energies [au] of all calculated compounds using different methods.

Molecule	E^a	E^b
trans,*trans*-**10**	-5531.830799	-5533.862983
cis,*trans*-**10**	-5531.819592	-5533.849799
cis,*cis*-**10**	-5531.821079	-5533.846703
trans-azobenzene	-572.777604	-572.987463
cis-azobenzene	-572.757313	-572.967326

[a] B3LYP-D3/6-31G*. [b] B3LYP-D3/def2-TZVP//B3LYP-D3/6-31G*.

Cartesian coordinates of the optimized geometry for *trans*,*trans*-**10** at B3LYP/6-31G* level of theory:

O	-5.34270500	2.44681800	-2.54570600
O	-8.08311900	5.04355000	0.16715100
N	-0.57830000	-3.41808500	1.45446800
N	-0.48995000	-3.43633800	0.19705000
N	-5.95614600	-1.81472200	-2.77718500
N	-7.55550400	-0.38379800	-2.19322000
N	-7.61401600	2.36323100	-2.25217200
N	-8.14236000	2.77521500	0.39653800
C	3.00703300	-2.90985800	3.63010800
C	3.01843900	-2.73661800	2.23565500
C	1.86810300	-2.92779600	1.48461100
C	0.66839100	-3.29423400	2.11979900
C	0.65200500	-3.48336700	3.50638600
C	1.81602700	-3.30069400	4.25156600
C	-1.72416600	-3.42759900	-0.49669500
C	-1.62653300	-3.60114000	-1.88310800
C	-2.76317000	-3.51123200	-2.68197500
C	-4.00994900	-3.23444900	-2.11118400
C	-4.10559000	-3.09061500	-0.71706400
C	-2.97962900	-3.18548500	0.08994300
C	-5.22954500	-3.07280600	-3.00105400
C	-7.24967200	-1.65830200	-2.31482200
C	-5.42514500	-0.54670600	-2.96109400
C	-6.43297900	0.31790000	-2.58393600
C	-6.38664800	1.79186500	-2.48005800
C	-7.76738800	3.74954100	-1.84742800
C	-8.01835700	3.92343000	-0.33013500
C	-8.18275600	2.75653000	1.86353100
C	-4.05888900	-0.27447100	-3.49650000
C	-8.82285700	4.52499900	-2.67594900
C	-10.22544300	3.90621300	-2.57953100
C	-8.37641000	4.66792100	-4.13655300
C	-9.60981300	2.55655500	2.44406600
C	-9.57462300	2.64466900	3.97760000
C	-10.60650100	3.56793400	1.86467700

O	-5.34215000	-2.44666600	2.54579000
O	-8.08261800	-5.04380200	-0.16716800
N	-0.57838400	3.41829600	-1.45449900
N	-0.49008800	3.43665200	-0.19707900
N	-5.95622300	1.81480600	2.77711600
N	-7.55534800	0.38362800	2.19312800
N	-7.61344400	-2.36338200	2.25202400
N	-8.14195400	-2.77547100	-0.39660000
C	3.00706400	2.90991500	-3.62992100
C	1.81616300	3.30101400	-4.25142700
C	0.65209700	3.48372300	-3.50633400
C	0.66832900	3.29438400	-2.11976900
C	1.86792800	2.92766100	-1.48453600
C	3.01831400	2.73643500	-2.23550300
C	-1.72433500	3.42790300	0.49662200
C	-1.62675600	3.60144100	1.88303700
C	-2.76342900	3.51152100	2.68185700
C	-4.01018000	3.23472200	2.11101600
C	-4.10576800	3.09090400	0.71688800
C	-2.97977600	3.18579200	-0.09006900
C	-5.22981000	3.07303800	3.00082700
C	-7.24970900	1.65818100	2.31469800
C	-5.42503100	0.54688000	2.96104200
C	-6.43272200	-0.31789400	2.58387900
C	-6.38617500	-1.79185300	2.48002100
C	-7.76661300	-3.74973200	1.84732700
C	-8.01773500	-3.92366800	0.33006900
C	-8.18248900	-2.75681800	-1.86359300
C	-4.05879700	0.27484900	3.49661400
C	-8.82183000	-4.52538700	2.67598500
C	-10.22456600	-3.90693300	2.57963100
C	-8.37523600	-4.66811400	4.13656200
C	-9.60963200	-2.55709700	-2.44400700
C	-9.57455100	-2.64530400	-3.97753800
C	-10.60613300	-3.56856300	-1.86445700
O	5.77205100	5.93243000	1.23628000
O	6.32716800	4.91104000	-2.89390700
O	4.07172300	-2.86313900	-4.39998400
O	5.77234700	-5.93242500	-1.23643300
O	6.32702600	-4.91098900	2.89380700
O	4.07180500	2.86323900	4.40046600
N	6.32150000	3.87169300	0.60260600
N	6.69738400	2.85352800	-1.95868100
N	6.29624000	-0.17369900	-3.40548700
N	4.88358500	1.36170600	-4.17858900
N	6.07950900	-2.91716800	-3.30239300
N	6.32152500	-3.87163400	-0.60269900
N	6.69707800	-2.85342900	1.95861100
N	6.29606600	0.17375400	3.40550500
N	4.88352500	-1.36163400	4.17885800
N	6.07924900	2.91720700	3.30224700
C	6.05494600	4.34563500	3.07966400
C	6.05943200	4.64675200	1.60558700
C	6.19363800	4.69040600	-0.52363900
C	5.85677500	5.95418700	-0.13384700
C	5.57908600	7.23130100	-0.83510200
C	6.41159700	4.18624900	-1.89617600
C	7.10146300	2.19269100	-3.20142600
C	8.55567100	1.65081000	-3.13390600
C	9.51848600	2.71041700	-2.57792000
C	9.02462300	1.17998200	-4.51979700
C	7.19354700	5.10798600	3.82596200
C	7.00700200	4.99325500	5.34392400
C	8.59018400	4.63772900	3.39635900
C	6.10838600	1.11542700	-3.57929100
C	5.17567800	-0.79676400	-3.91792300

C	4.28360400	0.13559100	-4.41040500
C	2.96378800	-0.03568000	-5.08630400
C	5.03241300	-2.26781100	-3.90589200
C	6.05523600	-4.34558400	-3.07979200
C	6.05964100	-4.64671400	-1.60570800
C	6.19363400	-4.69036100	0.52353000
C	5.85694000	-5.95417900	0.13370200
C	5.57936100	-7.23133700	0.83492000
C	6.41142300	-4.18618600	1.89608500
C	7.10124800	-2.19263100	3.20135900
C	8.55546600	-1.65077300	3.13377200
C	9.51823700	-2.71039400	2.57772600
C	9.02449500	-1.17996400	4.51964200
C	7.19388700	-5.10790800	-3.82603200
C	8.59049600	-4.63763300	-3.39636500
C	7.00741400	-4.99317400	-5.34400300
C	6.10821900	-1.11536800	3.57932600
C	5.17560100	0.79683400	3.91813700
C	4.28359300	-0.13551600	4.41075900
C	2.96395200	0.03577700	5.08700400
C	5.03232700	2.26787800	3.90607800
C	4.26265100	2.66601900	-4.45143200
C	4.26258700	-2.66597100	4.45166100
H	-8.40602400	1.73135300	-2.24585800
H	-8.00458800	1.88744400	-0.06924900
H	3.93532200	-2.42943600	1.74126900
H	1.86737400	-2.77791700	0.41089800
H	-0.28538000	-3.75545600	3.98195700
H	1.79055100	-3.43766300	5.33010700
H	-0.64633900	-3.77878700	-2.31466000
H	-2.67422700	-3.62637200	-3.75971500
H	-5.06388300	-2.87679100	-0.25341000
H	-3.06308600	-3.04675600	1.16185000
H	-5.93807100	-3.88770400	-2.83414800
H	-4.93145100	-3.11833300	-4.05411000
H	-6.79098000	4.21190100	-2.02407700
H	-7.82905500	3.74220400	2.18033700
H	-3.27630100	-0.61650100	-2.81096300
H	-3.89458800	-0.77562400	-4.45927700
H	-3.94972700	0.80212900	-3.63189700
H	-8.85937900	5.52095400	-2.21934100
H	-10.95229100	4.52460700	-3.11781000
H	-10.56685200	3.82686000	-1.54114600
H	-10.26065400	2.90586000	-3.03092000
H	-9.09863200	5.26667800	-4.70342100
H	-7.39932200	5.15888300	-4.20905600
H	-8.29534500	3.68939600	-4.62417200
H	-9.92900600	1.54417500	2.16482100
H	-8.90639300	1.89579800	4.41555000
H	-9.24056200	3.63745700	4.30737000
H	-10.57507400	2.48116500	4.39302900
H	-10.29389000	4.59860300	2.06590200
H	-11.59685100	3.41704700	2.30962400
H	-10.70686800	3.45767600	0.78117700
H	-8.40553900	-1.73161400	2.24566900
H	-8.00407100	-1.88769400	0.06914500
H	1.79081300	3.43817700	-5.32994700
H	-0.28520300	3.75602200	-3.98195400
H	1.86707400	2.77760900	-0.41084700
H	3.93510400	2.42904900	-1.74107400
H	-0.64658300	3.77908300	2.31463600
H	-2.67452800	3.62662700	3.75960300
H	-5.06403400	2.87707200	0.25318500
H	-3.06318600	3.04708000	-1.16198100
H	-5.93843300	3.88780500	2.83370100
H	-4.93180000	3.11879800	4.05389600

H	-6.79009300	-4.21189900	2.02386700
H	-7.82862300	-3.74242500	-2.18041700
H	-3.27616300	0.61734800	2.81136800
H	-3.89485500	0.77571000	4.45960800
H	-3.94937500	-0.80176700	3.63166800
H	-8.85815800	-5.52137600	2.21943400
H	-10.95122100	-4.52541800	3.11807000
H	-10.56609800	-3.82780200	1.54127000
H	-10.25995200	-2.90652700	3.03088700
H	-9.09723700	-5.26706800	4.70350400
H	-7.39799200	-5.15877100	4.20902100
H	-8.29443600	-3.68954300	4.62413500
H	-9.92894400	-1.54474400	-2.16479500
H	-8.90648100	-1.89634800	-4.41559000
H	-9.24035100	-3.63805800	-4.30727200
H	-10.57506000	-2.48199600	-4.39290600
H	-10.29343300	-4.59920600	-2.06567000
H	-11.59655100	-3.41780000	-2.30929700
H	-10.70638000	-3.45826300	-0.78095000
H	6.76355100	2.35175800	-1.07994600
H	6.75733400	-2.33868100	-2.82138000
H	6.76337000	-2.35168500	1.07986900
H	6.75695000	2.33872400	2.82105800
H	5.09834900	4.68941200	3.48926700
H	5.69671900	7.07196300	-1.90783900
H	6.26925300	8.01814400	-0.50725200
H	4.55910100	7.57772700	-0.62912200
H	7.07728700	2.98757600	-3.95350700
H	8.55432700	0.78258600	-2.46218500
H	9.51384100	3.61440100	-3.19998000
H	10.54160200	2.31814700	-2.56722400
H	9.26409100	3.00757500	-1.55613600
H	8.38853700	0.38359500	-4.91349000
H	9.02520400	2.01235500	-5.23605900
H	10.04941700	0.79600000	-4.46198800
H	7.08249000	6.16401500	3.54327500
H	7.09707700	3.95148600	5.67038600
H	7.76985500	5.57911500	5.86932600
H	6.02195300	5.35695500	5.65647100
H	8.75652600	3.59139000	3.67959400
H	9.36362200	5.23770700	3.88855800
H	8.73944500	4.72340200	2.31379700
H	2.79813500	-1.10197500	-5.24379500
H	2.93974200	0.47603800	-6.05743700
H	2.14095700	0.36605100	-4.48449900
H	5.09866500	-4.68938400	-3.48943500
H	5.69624600	-7.07183300	1.90771400
H	4.55967400	-7.57824100	0.62828800
H	6.27010200	-8.01791600	0.50763200
H	7.07709800	-2.98754500	3.95340900
H	8.55410500	-0.78254000	2.46206200
H	9.51358100	-3.61439800	3.19975700
H	10.54136400	-2.31815700	2.56701500
H	9.26381300	-3.00751200	1.55593600
H	9.02509300	-2.01233800	5.23590100
H	10.04929500	-0.79600500	4.46178600
H	8.38844600	-0.38356300	4.91336800
H	7.08283600	-6.16394000	-3.54335600
H	8.73972200	-4.72333300	-2.31379900
H	8.75684000	-3.59128400	-3.67956900
H	9.36396600	-5.23758000	-3.88855100
H	6.02237100	-5.35685500	-5.65659600
H	7.09752700	-3.95140900	-5.67046400
H	7.77027400	-5.57905600	-5.86937000
H	2.94043200	-0.47533400	6.05847800
H	2.79805700	1.10211600	5.24393700

H	2.14102900	-0.36659600	4.48576200
H	4.02558500	2.72190500	-5.51942700
H	5.00375600	3.44212700	-4.23839000
H	5.00371100	-3.44204900	4.23858500
H	4.02551800	-2.72188600	5.51965300

Cartesian coordinates of the optimized geometry for *cis*,*trans*-**10** at B3LYP/6-31G* level of theory:

O	4.13736700	1.64841100	3.36073400
O	5.60436300	5.10468700	0.36162000
N	0.54952000	-3.84921600	-1.64033700
N	0.45797800	-4.07427700	-0.40356500
N	5.61426700	-2.35788600	2.82052400
N	6.74533300	-0.55898500	2.16047500
N	6.14932900	2.11812700	2.36915600
N	6.33818300	3.02681700	-0.22114500
C	-2.99606400	-2.70602100	-3.61929900
C	-2.99339000	-2.82720400	-2.21975400
C	-1.86403500	-3.25919400	-1.54126200
C	-0.69582800	-3.56945400	-2.25957400
C	-0.69791800	-3.48492900	-3.65658500
C	-1.84524900	-3.06570200	-4.32950700
C	1.68602700	-4.13330200	0.29463200
C	1.58868500	-4.48703600	1.64720500
C	2.71439600	-4.41464600	2.46708500
C	3.93832300	-3.97079800	1.95431500
C	4.03109400	-3.66056300	0.58736800
C	2.92395100	-3.74025600	-0.24449600
C	5.13012400	-3.74725900	2.87123300
C	6.73180300	-1.87519000	2.16627100
C	4.88713600	-1.26008000	3.25730100
C	5.60308400	-0.16180700	2.82556100
C	5.21562800	1.26359200	2.90581800
C	5.88023400	3.53003500	2.16402700
C	5.92455600	3.96097900	0.68155300
C	6.27935100	3.25072800	-1.67102700
C	3.62740100	-1.35392400	4.05171100
C	6.74962600	4.47526800	3.03777600
C	8.25209400	4.32527200	2.76042400
C	6.43857700	4.27867100	4.52705100
C	7.68340000	3.46360200	-2.30316300
C	7.56081300	3.72237300	-3.81252500
C	8.44600800	4.60345800	-1.61736200
O	4.41943200	-2.28621200	-2.93216700
O	7.91642700	-4.66875300	-0.60163700
N	-1.68625300	5.51391300	-0.23523100
N	-1.11661800	5.55428400	-1.34265000
N	4.36032900	2.05213200	-2.92455400
N	6.04048100	0.83550100	-2.12046200
N	6.61450500	-1.82240500	-2.45543000
N	7.54804000	-2.50174300	0.00801800
C	-1.66531200	2.56291200	2.78053200
C	-1.47221600	3.91790600	3.08511000
C	-1.41170700	4.86943200	2.07577100
C	-1.49316600	4.47478200	0.73334700
C	-1.65654500	3.12053400	0.41399500
C	-1.74893100	2.17772400	1.43873900
C	0.00349000	4.73840400	-1.71278300
C	-0.01313300	4.15942000	-2.98745100
C	1.15154900	3.59607500	-3.49980900
C	2.35760000	3.64028200	-2.78518800
C	2.36027600	4.21623400	-1.50708300
C	1.19161500	4.74765300	-0.96577000
C	3.64554800	3.21925000	-3.46986800

C	5.57641100	2.05704400	-2.27088200
C	4.04248500	0.72781500	-3.19084100
C	5.09912900	-0.00216300	-2.68432800
C	5.31301300	-1.46573000	-2.71141400
C	7.04967500	-3.20012000	-2.31010900
C	7.55240000	-3.53206500	-0.88670000
C	7.77895900	-2.69006100	1.44579600
C	2.80875500	0.29624700	-3.90999700
C	8.08145000	-3.64955000	-3.37799600
C	9.37667700	-2.82577200	-3.33281700
C	7.46043100	-3.63570900	-4.78036900
C	9.21024300	-2.28995000	1.90022700
C	9.39700700	-2.59924400	3.39351000
C	10.29117800	-2.98299700	1.06166200
O	-5.17471800	5.86925300	0.48783900
O	-4.76827400	4.00334800	4.33082700
O	-1.89185600	-3.74142400	2.38825400
O	-5.12303700	-6.33528200	0.22997800
O	-6.72930900	-4.45079300	-3.27255300
O	-3.29788300	3.13094200	-2.96911000
N	-5.24531500	3.65903400	0.73203500
N	-5.07180400	2.13263900	3.04678100
N	-4.17650500	-1.10149900	3.36669500
N	-2.51169200	0.34475400	3.63916800
N	-4.16807100	-3.73785300	2.65344800
N	-5.48373200	-4.14188600	0.13804600
N	-6.56159800	-2.61272900	-1.91782700
N	-5.89866600	0.61579900	-2.66908900
N	-4.67358200	-0.87315500	-3.77857100
N	-5.41299200	3.25775000	-2.09557900
C	-5.29477800	4.61756600	-1.61582500
C	-5.26081100	4.64241100	-0.10901500
C	-5.11683000	4.26516300	1.98468800
C	-5.07402700	5.62053900	1.83290100
C	-4.94537900	6.77715500	2.75218600
C	-4.97508100	3.48127600	3.23110000
C	-4.88250300	1.17841900	4.13727500
C	-6.20936800	0.54644600	4.64322400
C	-7.29402400	1.61454700	4.84148400
C	-5.97261900	-0.22829700	5.94927400
C	-6.38467300	5.56447500	-2.19727900
C	-6.21967500	5.70814500	-3.71518900
C	-7.80658900	5.11869000	-1.82617700
C	-3.87883600	0.12887300	3.71815600
C	-2.97999100	-1.71673400	3.05795300
C	-1.92665500	-0.83657300	3.21720400
C	-0.45493900	-1.00997500	3.03417900
C	-2.93525700	-3.14002100	2.66233600
C	-4.36119300	-5.09714500	2.20409400
C	-5.01534100	-5.11847600	0.84623900
C	-5.92355900	-4.73943000	-1.04684700
C	-5.70099000	-6.08441300	-0.99029200
C	-5.93091900	-7.22677000	-1.90783700
C	-6.45250900	-3.94766700	-2.17867700
C	-6.96802500	-1.63920100	-2.93348300
C	-8.33947200	-0.98003900	-2.62579900
C	-9.38296700	-2.03543600	-2.23247000
C	-8.82993500	-0.16493600	-3.83291200
C	-5.12910000	-5.97836100	3.23387900
C	-6.52997600	-5.43046300	3.54322300
C	-4.30152100	-6.15606600	4.51274400
C	-5.86693600	-0.61782800	-3.12147500
C	-4.70075900	1.19318000	-3.04080700
C	-3.92298200	0.28944100	-3.73786100
C	-2.58416700	0.44266000	-4.37977200
C	-4.38372400	2.59858500	-2.70620800

C	-1.79157700	1.58166300	3.93698200
C	-4.21209600	-2.15070500	-4.34393100
H	7.04376100	1.70964300	2.12644200
H	6.41470500	2.06290200	0.07976900
H	-3.87647700	-2.55268100	-1.65315000
H	-1.84832200	-3.33118900	-0.45858800
H	0.21688100	-3.71872300	-4.19290400
H	-1.83143600	-2.98041600	-5.41377000
H	0.61322100	-4.75679200	2.04088900
H	2.62761600	-4.66189800	3.52276400
H	4.97291400	-3.30766900	0.17931700
H	2.99511500	-3.45015600	-1.28747700
H	5.96751400	-4.39455200	2.59939500
H	4.86367100	-3.98231600	3.90640000
H	4.83805700	3.66537300	2.47042100
H	5.69788900	4.16735100	-1.80679700
H	2.82532500	-1.84354000	3.48859500
H	3.77745600	-1.92475300	4.97796500
H	3.30992200	-0.34190600	4.30544500
H	6.44710900	5.48762300	2.74492200
H	8.82514900	5.03502600	3.36758600
H	8.49570800	4.52047300	1.71013000
H	8.61076300	3.31970000	3.01622600
H	6.99597900	5.00156800	5.13402800
H	5.37097000	4.40956700	4.73448500
H	6.72028000	3.27336100	4.86131500
H	8.23975300	2.52747700	-2.15910400
H	7.05992100	2.89805400	-4.33216400
H	6.99921200	4.64527200	-4.01074000
H	8.55249700	3.84244500	-4.26219000
H	7.89465200	5.54858300	-1.68398700
H	9.42372100	4.74541700	-2.09208200
H	8.61573100	4.39068700	-0.55844300
H	7.27766500	-1.06066200	-2.37911800
H	7.14032800	-1.61898700	-0.27355100
H	-1.42617800	4.23645600	4.12331900
H	-1.32993600	5.92624500	2.31232300
H	-1.78431400	2.82157900	-0.62156200
H	-1.92637400	1.13724700	1.18231100
H	-0.93873900	4.16165400	-3.55373500
H	1.13388400	3.15690600	-4.49479700
H	3.28380300	4.29773100	-0.93883400
H	1.21403600	5.22332200	0.00941400
H	4.35598900	4.04909100	-3.45733400
H	3.43995400	3.00475600	-4.52315200
H	6.14497100	-3.80230300	-2.44518700
H	7.65400700	-3.76133300	1.63136300
H	1.90319500	0.64692900	-3.40194900
H	2.77929500	0.67935300	-4.93946500
H	2.79172600	-0.79422700	-3.94183900
H	8.32765600	-4.68366600	-3.11032400
H	10.09827000	-3.20948700	-4.06265400
H	9.85285100	-2.86840600	-2.34667800
H	9.19612700	-1.77215500	-3.58386800
H	8.17032700	-4.02582000	-5.51886700
H	6.55363600	-4.24912700	-4.82322800
H	7.18805300	-2.61791300	-5.08378500
H	9.29466500	-1.20449500	1.76024300
H	8.66567600	-2.07273100	4.01588800
H	9.30043000	-3.67580400	3.58775900
H	10.39581600	-2.29488400	3.72503800
H	10.20687700	-4.07370000	1.12133600
H	11.28648100	-2.69574700	1.42019600
H	10.22217800	-2.70242700	0.00675000
H	-5.20333600	1.80103700	2.09820400
H	-4.96313300	-3.12180800	2.77427000

H	-6.29696800	-2.29330900	-0.99276900
H	-6.20166200	2.68699600	-1.81499300
H	-4.31714900	4.96977700	-1.96664500
H	-4.06401200	7.37824200	2.49844000
H	-4.84787600	6.40127900	3.77171100
H	-5.82261500	7.43283500	2.68875500
H	-4.47237600	1.77820800	4.95814600
H	-6.54857600	-0.16469400	3.87943700
H	-6.96809700	2.38585400	5.54910000
H	-8.20593900	1.15322700	5.23764900
H	-7.55059400	2.11208600	3.90169700
H	-5.23751100	-1.02734500	5.82068100
H	-5.62055100	0.44315400	6.74377300
H	-6.90727000	-0.68378000	6.29586100
H	-6.20314900	6.54448000	-1.73750100
H	-6.37595000	4.74829900	-4.21996300
H	-6.94902800	6.42192200	-4.11477600
H	-5.21727500	6.06577800	-3.97621500
H	-8.05414800	4.15300800	-2.28411900
H	-8.54170900	5.84675600	-2.18663100
H	-7.93678200	5.02422800	-0.74168000
H	-0.27209400	-2.00156700	2.61858900
H	0.08356500	-0.93095500	3.98835300
H	-0.03388000	-0.25698300	2.35733200
H	-3.35286800	-5.51113100	2.08587300
H	-6.36131600	-6.84635200	-2.83531600
H	-4.98995300	-7.74373800	-2.13106800
H	-6.61489200	-7.95985000	-1.46279200
H	-7.08444000	-2.23118900	-3.84734400
H	-8.19527600	-0.29090600	-1.78391300
H	-9.51414900	-2.77897900	-3.02832200
H	-10.35282600	-1.55665400	-2.05637600
H	-9.10209800	-2.56997500	-1.32025500
H	-8.97609200	-0.81185300	-4.70815700
H	-9.79429900	0.30394400	-3.60676400
H	-8.12537700	0.62643800	-4.10071600
H	-5.24393900	-6.96160300	2.75825300
H	-7.12954100	-5.29796500	2.63506900
H	-6.47425400	-4.46315900	4.05737600
H	-7.07150300	-6.11778900	4.20239700
H	-3.31974500	-6.59289100	4.29704600
H	-4.13875300	-5.19396300	5.01066400
H	-4.81918900	-6.81764800	5.21647800
H	-2.61095100	0.15718300	-5.43956200
H	-2.28624100	1.48833300	-4.29988200
H	-1.82410200	-0.17855900	-3.89340500
H	-0.79884400	1.29529900	4.30104300
H	-2.29922800	2.07678100	4.76973200
H	-5.04631800	-2.85626900	-4.29823200
H	-3.97692100	-1.99677100	-5.40279700

Cartesian coordinates of the optimized geometry for *cis*,*cis*-**10** at B3LYP/6-31G* level of theory:

O	3.82652900	-2.89332300	-1.96642100
O	6.53038000	-4.94421500	1.11766200
N	-0.40510500	5.64899900	0.64414800
N	0.13562900	5.70010800	-0.47574400
N	4.32050200	1.25379100	-3.03423300
N	5.97202700	0.00561900	-2.22391200
N	6.09483000	-2.68957600	-1.70510500
N	6.61127600	-2.66827400	0.98341300
C	-1.83235500	2.55934400	3.12599900
C	-1.98129800	2.44445900	1.74155900
C	-1.40456800	3.37853200	0.87259300

C	-0.69233200	4.46473000	1.39860400
C	-0.51771500	4.57079900	2.78833500
C	-1.05347800	3.61199400	3.63369100
C	0.83941800	4.63795000	-1.12826100
C	0.68018900	4.56968400	-2.52134100
C	1.52251600	3.76883100	-3.27984700
C	2.58091700	3.06796200	-2.68072200
C	2.72479700	3.12883900	-1.29148700
C	1.85504900	3.89434700	-0.51097700
C	3.59132200	2.39207200	-3.59491300
C	5.63352300	1.21912600	-2.60521400
C	3.81010200	-0.02781800	-2.91188800
C	4.85164300	-0.77998000	-2.40722500
C	4.85155900	-2.20766000	-2.02527300
C	6.29712300	-3.98813200	-1.08581300
C	6.49294400	-3.91765000	0.44794300
C	6.54796900	-2.40735100	2.42675600
C	2.41819800	-0.39683100	-3.29636600
C	7.42512000	-4.81884100	-1.74843100
C	8.78771900	-4.11011400	-1.70742000
C	7.04755400	-5.21690800	-3.18101200
C	7.93696600	-2.15557100	3.07382500
C	7.78829200	-1.95656200	4.59004600
C	8.92162200	-3.29109700	2.76898500
O	3.82711400	2.89339000	1.96620600
O	6.53242100	4.94334300	-1.11728600
N	-0.40473700	-5.64923300	-0.64544000
N	0.13585000	-5.70051900	0.47451200
N	4.31944100	-1.25386000	3.03427100
N	5.97155300	-0.00630100	2.22419900
N	6.09539500	2.68884000	1.70535000
N	6.61254100	2.66736800	-0.98299600
C	-1.83297300	-2.55945300	-3.12655800
C	-1.05328100	-3.61141800	-3.63441000
C	-0.51724900	-4.57024800	-2.78924500
C	-0.69235000	-4.46480700	-1.39952700
C	-1.40530300	-3.37917800	-0.87331100
C	-1.98235000	-2.44514800	-1.74210600
C	0.83910800	-4.63825700	1.12745600
C	0.67967900	-4.57036800	2.52052200
C	1.52163500	-3.76938200	3.27930800
C	2.57983700	-3.06797200	2.68047200
C	2.72391100	-3.12849000	1.29124400
C	1.85455900	-3.89417000	0.51046400
C	3.58982200	-2.39184800	3.59495900
C	5.63254900	-1.21967500	2.60547000
C	3.80952200	0.02792700	2.91179600
C	4.85141200	0.77970400	2.40729400
C	4.85189300	2.20737400	2.02529600
C	6.29825700	3.98733900	1.08612700
C	6.49442000	3.91679100	-0.44757700
C	6.54935500	2.40645800	-2.42635200
C	2.41767600	0.39743600	3.29599300
C	7.42635500	4.81769600	1.74901900
C	8.78876100	4.10858400	1.70824300
C	7.04861100	5.21580500	3.18153800
C	7.93837600	2.15416600	-3.07318200
C	7.78989300	1.95524300	-4.58942900
C	8.92339300	3.28931600	-2.76812700
O	-4.47504900	-6.06934000	-0.20047100
O	-4.91580300	-4.16368600	-4.03994000
O	-2.50740400	3.55183900	-2.43199400
O	-4.47335100	6.06995800	0.20067100
O	-4.91408200	4.16435300	4.04015900
O	-2.50870700	-3.55221400	2.43127500
N	-5.19612300	-3.97649000	-0.41168700

N	-5.50984700	-2.43094700	-2.66208000
N	-4.88777500	0.88891500	-3.07890900
N	-3.25619100	-0.51364200	-3.64268800
N	-4.79352600	3.56879400	-2.43877500
N	-5.19431100	3.97706300	0.41188500
N	-5.50858400	2.43170900	2.66239100
N	-4.88780700	-0.88833500	3.07896400
N	-3.25551900	0.51352500	3.64245400
N	-4.79482200	-3.56827200	2.43896600
C	-4.89467900	-4.91811100	1.92311600
C	-4.88389400	-4.92059000	0.41527600
C	-4.96999700	-4.52341900	-1.67758600
C	-4.52260300	-5.80621500	-1.54741000
C	-4.06903900	-6.86338100	-2.48286200
C	-5.13109000	-3.71942600	-2.90780500
C	-5.64444000	-1.42512500	-3.71225100
C	-7.08870600	-0.87181800	-3.84162400
C	-8.11574700	-2.01305200	-3.84702400
C	-7.23110400	-0.01737500	-5.11135100
C	-6.11494200	-5.68739500	2.50447600
C	-5.93173100	-5.93993700	4.00692000
C	-7.45337800	-4.99305500	2.20901200
C	-4.62231500	-0.33163400	-3.48627500
C	-3.67066500	1.53452400	-2.97754700
C	-2.64044100	0.68206400	-3.32359100
C	-1.16617100	0.88436900	-3.40369400
C	-3.58014100	2.95531700	-2.58761500
C	-4.89268700	4.91866900	-1.92291700
C	-4.88208600	4.92116500	-0.41507500
C	-4.96828400	4.52402600	1.67778900
C	-4.52095400	5.80683700	1.54762500
C	-4.06761500	6.86409400	2.48307400
C	-5.12951500	3.72011200	2.90805500
C	-5.64334100	1.42598200	3.71262400
C	-7.08779700	0.87328100	3.84245400
C	-8.11434900	2.01495500	3.84836200
C	-7.23007700	0.01873800	5.11212100
C	-6.11244000	5.68868200	-2.50440000
C	-7.45130400	4.99513200	-2.20902800
C	-5.92896300	5.94107300	-4.00683300
C	-4.62175200	0.33207700	3.48634400
C	-3.67098300	-1.53442000	2.97723400
C	-2.64032900	-0.68240800	3.32309400
C	-1.16610900	-0.88528000	3.40249600
C	-3.58114500	-2.95525600	2.58730200
C	-2.56837700	-1.70020300	-4.14671900
C	-2.56711900	1.69979300	4.14636300
H	6.86752100	-2.04541000	-1.82749500
H	6.48838000	-1.86986400	0.37431800
H	-2.58561100	1.64266700	1.32703400
H	-1.58064600	3.30953800	-0.19714300
H	0.01413500	5.42959500	3.18713300
H	-0.92244400	3.71507500	4.70884600
H	-0.11003600	5.14872100	-2.98674000
H	1.38657300	3.72447300	-4.35858300
H	3.53063800	2.59693500	-0.79652600
H	2.02028800	3.94758700	0.55967800
H	4.34000100	3.12259300	-3.91614800
H	3.09073400	2.04887200	-4.50649200
H	5.35342100	-4.52601300	-1.21754800
H	6.12262100	-3.31186200	2.87346200
H	1.68490500	0.21766200	-2.76279900
H	2.24368900	-0.25994900	-4.37250000
H	2.25190600	-1.44521600	-3.04594200
H	7.49608800	-5.72699800	-1.13835800
H	9.57097200	-4.76826600	-2.09936700

H	9.07324600	-3.82900700	-0.68730900
H	8.79405600	-3.20346000	-2.32699400
H	7.82548800	-5.84997600	-3.62308800
H	6.10461400	-5.77487000	-3.20422300
H	6.92990900	-4.33416300	-3.82050300
H	8.32458200	-1.22417100	2.64069100
H	7.13016600	-1.11468600	4.83066200
H	7.38143200	-2.85799700	5.06762600
H	8.76349800	-1.75658600	5.04734600
H	8.54967900	-4.25285100	3.13903700
H	9.88745000	-3.08961600	3.24671500
H	9.09464000	-3.39634500	1.69428700
H	6.86783800	2.04441000	1.82791700
H	6.48901300	1.86903200	-0.37393000
H	-0.92180600	-3.71398000	-4.70956100
H	0.01522900	-5.42858600	-3.18819300
H	-1.58163800	-3.31058800	0.19641300
H	-2.58719400	-1.64381900	-1.32746400
H	-0.11037800	-5.14978900	2.98572900
H	1.38554800	-3.72534300	4.35804100
H	3.52963600	-2.59619700	0.79651300
H	2.01998900	-3.94716600	-0.56017200
H	4.33823900	-3.12233000	3.91690300
H	3.08878200	-2.04823600	4.50613500
H	5.35469300	4.52551500	1.21764100
H	6.12442900	3.31112700	-2.87313800
H	2.25173000	1.44581700	3.04532700
H	1.68429000	-0.21695400	2.76244100
H	2.24295900	0.26084500	4.37213200
H	7.49770600	5.72586000	1.13899900
H	9.57211700	4.76647300	2.10042900
H	9.07442600	3.82749800	0.68816500
H	8.79470100	3.20186900	2.32773000
H	7.82661000	5.84867400	3.62378400
H	6.10579700	5.77398700	3.20459300
H	6.93063500	4.33306900	3.82098300
H	8.32555700	1.22261300	-2.63998900
H	7.13149000	1.11362200	-4.83018200
H	7.38345500	2.85684100	-5.06706300
H	8.76510200	1.75490900	-5.04656600
H	8.55187000	4.25122300	-3.13820400
H	9.88923300	3.08750400	-3.24569500
H	9.09625900	3.39445300	-1.69339300
H	-5.61906300	-2.16187500	-1.69065000
H	-5.60183800	2.95841200	-2.42370900
H	-5.61783200	2.16260100	1.69097500
H	-5.60290200	-2.95757700	2.42417500
H	-3.98192200	-5.43399500	2.23788500
H	-3.02451700	-7.12799400	-2.27858800
H	-4.15610200	-6.48661200	-3.50304500
H	-4.67323100	-7.77268700	-2.37789700
H	-5.41269500	-1.96280500	-4.63838400
H	-7.27757300	-0.22768300	-2.97303200
H	-7.92959700	-2.70864000	-4.67487800
H	-9.12655200	-1.60802800	-3.97029000
H	-8.09382700	-2.58902900	-2.91747300
H	-6.54241400	0.83104000	-5.10839900
H	-7.03919000	-0.61872900	-6.00995300
H	-8.25123500	0.37512500	-5.19155800
H	-6.11761200	-6.65886600	1.99140300
H	-5.88077800	-4.99602500	4.56065500
H	-6.77139300	-6.52090700	4.40469500
H	-5.01009600	-6.49811600	4.20817400
H	-7.55325700	-4.05531400	2.77016500
H	-8.28784000	-5.63567100	2.51031700
H	-7.57023100	-4.76486400	1.14411600

H	-0.93115500	1.90343700	-3.09620800
H	-0.79361400	0.73253600	-4.42616000
H	-0.63311400	0.18020000	-2.75442000
H	-3.97959500	5.43401600	-2.23760100
H	-4.15459700	6.48731800	3.50326200
H	-3.02315200	7.12893800	2.27881600
H	-4.67200000	7.77327300	2.37809200
H	-5.41108700	1.96357900	4.63867800
H	-7.27725500	0.22933600	2.97385000
H	-7.92761500	2.71036000	4.67623700
H	-9.12528200	1.61034200	3.97192000
H	-8.09249600	2.59103300	2.91887200
H	-7.03755000	0.61988400	6.01073200
H	-8.25034800	-0.37332600	5.19268100
H	-6.54175700	-0.82997800	5.10879500
H	-6.11457600	6.66016500	-1.99135000
H	-7.56842500	4.76711600	-1.14412200
H	-7.55163600	4.05738700	-2.77009300
H	-8.28537200	5.63818300	-2.51049500
H	-5.00696000	6.49866200	-4.20803700
H	-5.87856800	4.99711700	-4.56054400
H	-6.76822900	6.52256700	-4.40468100
H	-0.79277700	-0.73248400	4.42452600
H	-0.93173700	-1.90475600	3.09587200
H	-0.63317000	-0.18206600	2.75207800
H	-1.86951400	-1.37401400	-4.92563800
H	-3.31514500	-2.33136100	-4.63745500
H	-3.31338700	2.33084000	4.63800200
H	-1.86769200	1.37315200	4.92458700

Cartesian coordinates of the optimized geometry for *trans*-azobenzene at B3LYP/6-31G* level of theory:

C	-4.01409500	-1.09454000	0.00005400
C	-2.63333400	-1.28650500	0.00001400
C	-1.76803000	-0.18447000	-0.00009800
C	-2.29375000	1.11920500	-0.00004000
C	-3.67125300	1.30357200	0.00004900
C	-4.53498700	0.20045400	0.00005700
H	-4.68130700	-1.95202600	0.00008700
H	-2.19790100	-2.28120000	0.00000700
H	-1.60832500	1.95928700	-0.00007600
H	-4.08065600	2.31052700	0.00007400
H	-5.61077300	0.35465700	0.00011100
N	-0.38470400	-0.49932500	-0.00012800
N	0.38471700	0.49936700	-0.00004700
C	1.76803400	0.18448700	0.00000100
C	2.63335700	1.28651100	-0.00001800
C	2.29372600	-1.11919300	0.00001600
C	4.01411200	1.09452100	0.00005500
H	2.19793000	2.28120900	-0.00002900
C	3.67122900	-1.30358800	0.00001100
H	1.60829100	-1.95926700	-0.00000300
C	4.53498000	-0.20048900	0.00004700
H	4.68134900	1.95198600	0.00008400
H	4.08060100	-2.31055500	0.00001300
H	5.61076500	-0.35470300	0.00006900

Cartesian coordinates of the optimized geometry for *cis*-azobenzene at B3LYP/6-31G* level of theory:

C	-3.44347600	-0.36091800	0.68464500
C	-2.54636800	0.70062500	0.78084900
C	-1.43153700	0.75490300	-0.06641900

C	-1.26046500	-0.22021400	-1.06029900
C	-2.18419600	-1.25727000	-1.17409900
C	-3.26725400	-1.34135700	-0.29517300
H	-4.29281700	-0.41132700	1.36050100
H	-2.68920600	1.49373600	1.50896100
H	-0.42024000	-0.15557200	-1.74350000
H	-2.05533900	-2.00520500	-1.95186900
H	-3.97901200	-2.15723000	-0.38465800
N	-0.62458900	1.93982800	0.01518800
N	0.62450400	1.93984000	-0.01534800
C	1.43148800	0.75494800	0.06639500
C	2.54612600	0.70048900	-0.78111400
C	1.26067700	-0.21992800	1.06056200
C	3.44328700	-0.36100400	-0.68485400
H	2.68877200	1.49342300	-1.50945800
C	2.18446500	-1.25692500	1.17440900
H	0.42062000	-0.15513600	1.74395100
C	3.26732000	-1.34120100	0.29525000
H	4.29247200	-0.41155600	-1.36089500
H	2.05581800	-2.00466600	1.95240100
H	3.97912400	-2.15703100	0.38477500

Cartesian coordinates of the optimized geometry for *trans*,*trans*-**10** at B3LYP-D3/6-31G* level of theory:

O	-4.77976300	3.81325700	-0.92528200
O	-7.35501200	3.56414000	3.13893800
N	-0.65825200	-1.71851300	0.69003500
N	-0.46986300	-1.69359200	-0.55643200
N	-5.85120400	0.22216100	-2.99532100
N	-7.25281400	1.31720100	-1.66352600
N	-7.02967600	3.79773300	-0.47047000
N	-7.60598600	2.00178500	1.49934800
C	2.52335400	-0.82456100	3.28039800
C	2.81259300	-1.05482200	1.92419500
C	1.80971200	-1.41009900	1.03173500
C	0.48827900	-1.54990300	1.49443300
C	0.21638200	-1.41578600	2.86181000
C	1.22466000	-1.04564300	3.74494900
C	-1.67445600	-1.70491900	-1.30648500
C	-1.54684300	-1.49682400	-2.68474200
C	-2.68330800	-1.30507500	-3.47050200
C	-3.95462900	-1.32417100	-2.89101300
C	-4.07525400	-1.62300900	-1.52482200
C	-2.95502400	-1.81923600	-0.73600100
C	-5.18715400	-0.90831100	-3.67191800
C	-7.07502700	0.20889500	-2.35329800
C	-5.22695700	1.41888100	-2.68345100
C	-6.11475300	2.08072300	-1.86185700
C	-5.89224600	3.31347000	-1.08688600
C	-6.96038200	4.32400100	0.88503100
C	-7.33234400	3.26357600	1.94583000
C	-8.05331600	0.93855100	2.40111000
C	-3.87287400	1.82459000	-3.15938100
C	-7.78849700	5.61372100	1.08469500
C	-9.28364600	5.38192600	0.82657000
C	-7.23491500	6.73966800	0.20453600
C	-9.49978700	0.47479900	2.08976700
C	-10.00976800	-0.46401800	3.19297100
C	-10.43693300	1.67745700	1.92351600
O	-4.77941500	-3.81330700	0.92547800
O	-7.35460800	-3.56423100	-3.13894300
N	-0.65837000	1.71852700	-0.69012300
N	-0.46993400	1.69360700	0.55633600
N	-5.85112500	-0.22220700	2.99538800

N	-7.25261500	-1.31731400	1.66351200
N	-7.02929400	-3.79782200	0.47047800
N	-7.60587300	-2.00193700	-1.49933800
C	2.52323800	0.82457200	-3.28048200
C	1.22445300	1.04516900	-3.74497900
C	0.21616100	1.41534600	-2.86186800
C	0.48815000	1.54996200	-1.49456400
C	1.80966600	1.41064200	-1.03193700
C	2.81255500	1.05533900	-1.92437800
C	-1.67448600	1.70501300	1.30643700
C	-1.54682700	1.49662200	2.68464800
C	-2.68326600	1.30486800	3.47042800
C	-3.95461500	1.32424400	2.89101900
C	-4.07529000	1.62339300	1.52490100
C	-2.95507000	1.81964000	0.73605500
C	-5.18714700	0.90837100	3.67191100
C	-7.07491700	-0.20900000	2.35330000
C	-5.22679300	-1.41889000	2.68354200
C	-6.11452400	-2.08077400	1.86190200
C	-5.89191700	-3.31352800	1.08698100
C	-6.95992400	-4.32405800	-0.88503200
C	-7.33200400	-3.26367600	-1.94583100
C	-8.05332400	-0.93874600	-2.40109100
C	-3.87273000	-1.82456500	3.15957300
C	-7.78789500	-5.61387600	-1.08471300
C	-9.28306900	-5.38224900	-0.82658300
C	-7.23418800	-6.73976700	-0.20455900
C	-9.49983000	-0.47513000	-2.08969800
C	-10.00990400	0.46370900	-3.19283900
C	-10.43687800	-1.67787300	-1.92350700
O	5.65221400	5.87347600	-1.86301400
O	5.74885100	2.74140700	-4.79329300
O	3.79171200	-4.60976300	-1.62121300
O	5.65244200	-5.87338500	1.86302600
O	5.74913300	-2.74134600	4.79333300
O	3.79149900	4.60962500	1.62103300
N	6.15545400	3.77324900	-1.32476500
N	6.26234000	1.51480100	-2.92842500
N	6.00903100	-1.91802500	-2.61478700
N	4.33534200	-0.81831800	-3.57686900
N	6.06987200	-4.42009000	-1.46297000
N	6.15567100	-3.77315500	1.32479000
N	6.26244900	-1.51469400	2.92844500
N	6.00899300	1.91808600	2.61475000
N	4.33538400	0.81836900	3.57696700
N	6.06969200	4.42018600	1.46298100
C	6.18117000	5.50485400	0.51148000
C	6.00072900	4.98111500	-0.88685600
C	5.88181200	3.85376800	-2.69197700
C	5.57282200	5.14116900	-3.02226900
C	5.19036100	5.83620900	-4.27399900
C	5.95951000	2.67756400	-3.57645700
C	6.62463600	0.29231400	-3.64671100
C	8.11521900	-0.08320900	-3.44507600
C	9.01340700	1.14888700	-3.62938400
C	8.52964800	-1.19827000	-4.41765900
C	7.51120000	6.29064900	0.66468500
C	7.54314400	7.02565200	2.01008400
C	8.74494800	5.39273900	0.48742900
C	5.68377900	-0.82532100	-3.26810400
C	4.84381300	-2.65291700	-2.49440900
C	3.78529500	-1.98717300	-3.07945300
C	2.34217500	-2.35037200	-3.19320300
C	4.82116400	-3.96567000	-1.82555600
C	6.18138200	-5.50475600	-0.51146500
C	6.00094600	-4.98102000	0.88687200

C	5.88203700	-3.85368300	2.69200200
C	5.57306500	-5.14109000	3.02229100
C	5.19065400	-5.83614600	4.27402600
C	5.95971300	-2.67747800	3.57648500
C	6.62471400	-0.29219500	3.64672300
C	8.11527600	0.08338200	3.44504700
C	9.01351400	-1.14865600	3.62944700
C	8.52967600	1.19854500	4.41752600
C	7.51142100	-6.29052800	-0.66468300
C	8.74515600	-5.39259100	-0.48746400
C	7.54335000	-7.02554800	-2.01007200
C	5.68380400	0.82540200	3.26813000
C	4.84373600	2.65291600	2.49436900
C	3.78527700	1.98718200	3.07951800
C	2.34212700	2.35026700	3.19322400
C	4.82100500	3.96565200	1.82547700
C	3.56956500	0.25301400	-4.21801600
C	3.56962000	-0.25302400	4.21802400
H	-7.88542800	3.32342600	-0.73297800
H	-7.55765800	1.77070100	0.50991400
H	3.82645700	-0.90896000	1.56341200
H	2.02423500	-1.54463900	-0.02190800
H	-0.80745700	-1.53844700	3.19792700
H	0.99071900	-0.87981600	4.79403400
H	-0.55326800	-1.42012900	-3.11223900
H	-2.57500700	-1.08468200	-4.52996500
H	-5.05866800	-1.65653700	-1.06919900
H	-3.05987500	-2.05026900	0.31463900
H	-5.90746900	-1.72674500	-3.75197200
H	-4.91861400	-0.59953900	-4.68728200
H	-5.90644500	4.56376100	1.06047600
H	-8.04926500	1.38621300	3.39945800
H	-3.08523200	1.33175500	-2.57890400
H	-3.72097200	1.56868300	-4.21414400
H	-3.75841800	2.90061100	-3.02201600
H	-7.66061400	5.88275700	2.13941800
H	-9.85050900	6.30336400	0.99922100
H	-9.69278100	4.61146900	1.48935300
H	-9.46563000	5.07970800	-0.21312700
H	-7.78829700	7.67026700	0.37467200
H	-6.17613000	6.92744300	0.41606900
H	-7.32011900	6.47959100	-0.85646500
H	-9.47450600	-0.08606000	1.14678000
H	-9.37177300	-1.34540500	3.30036800
H	-10.04705000	0.05690000	4.15862900
H	-11.02443100	-0.80719100	2.96253900
H	-10.41659500	2.31788600	2.81411300
H	-11.46755600	1.33689200	1.77495300
H	-10.15962600	2.29301100	1.06321600
H	-7.88508300	-3.32356200	0.73294500
H	-7.55754000	-1.77084300	-0.50990700
H	0.99045800	0.87893100	-4.79398800
H	-0.80773600	1.53761600	-3.19793700
H	2.02425800	1.54560400	0.02163600
H	3.82649800	0.90987400	-1.56365200
H	-0.55324500	1.41967000	3.11207700
H	-2.57493600	1.08423200	4.52983700
H	-5.05872400	1.65718400	1.06934500
H	-3.05996100	2.05088400	-0.31453400
H	-5.90750000	1.72677100	3.75191400
H	-4.91862200	0.59967300	4.68730300
H	-5.90596100	-4.56370200	-1.06046900
H	-8.04926200	-1.38641300	-3.39943400
H	-3.08505600	-1.33202300	2.57888000
H	-3.72076000	-1.56826700	4.21422800
H	-3.75838000	-2.90064700	3.02260700

H	-7.65997800	-5.88289000	-2.13943700
H	-9.84983500	-6.30373700	-0.99928100
H	-9.69228600	-4.61179800	-1.48932400
H	-9.46508500	-5.08010100	0.21312800
H	-7.78745800	-7.67042900	-0.37471200
H	-6.17537800	-6.92741200	-0.41608000
H	-7.31943500	-6.47970900	0.85644300
H	-9.47458000	0.08567600	-1.14667900
H	-9.37198300	1.34515600	-3.30018900
H	-10.04715400	-0.05715100	-4.15852900
H	-11.02459300	0.80678700	-2.96237600
H	-10.41653000	-2.31822400	-2.81416000
H	-11.46752200	-1.33739700	-1.77488100
H	-10.15949400	-2.29348100	-1.06327200
H	6.46074000	1.58160800	-1.93607200
H	6.79578400	-3.71231200	-1.47034200
H	6.46081600	-1.58149200	1.93608500
H	6.79567100	3.71247800	1.47041400
H	5.34692300	6.18462200	0.70997500
H	5.18968600	5.10842800	-5.08682400
H	5.89644700	6.64242800	-4.50653200
H	4.19302100	6.28293400	-4.18239700
H	6.48135600	0.53936100	-4.70450900
H	8.22794900	-0.46051300	-2.41997700
H	8.86506500	1.59470900	-4.62094800
H	10.06748600	0.86327200	-3.54241400
H	8.81268700	1.92383500	-2.88321600
H	7.93009700	-2.09968900	-4.27380100
H	8.41747600	-0.86453000	-5.45771600
H	9.58309100	-1.46015300	-4.26686800
H	7.51128400	7.03617000	-0.14263300
H	7.49052400	6.31172800	2.83901400
H	8.46829800	7.60383800	2.11294800
H	6.69744900	7.71616400	2.10708900
H	8.84230800	4.68448600	1.31958900
H	9.65715700	5.99883500	0.47113700
H	8.70168100	4.81735900	-0.44396000
H	2.23092100	-3.41091200	-2.96545600
H	1.95285700	-2.14731500	-4.19834200
H	1.72688900	-1.78830400	-2.48002100
H	5.34714300	-6.18453500	-0.70995800
H	5.18979300	-5.10832900	5.08681800
H	4.19341200	-6.28307900	4.18237700
H	5.89688900	-6.64220900	4.50665000
H	6.48147300	-0.53923300	4.70452600
H	8.22797600	0.46060400	2.41991200
H	8.86519600	-1.59441000	4.62104600
H	10.06758500	-0.86301800	3.54245000
H	8.81281800	-1.92367300	2.88334300
H	8.41752800	0.86488900	5.45761200
H	9.58311000	1.46044500	4.26669500
H	7.93010000	2.09993600	4.27359600
H	7.51153500	-7.03603900	0.14264600
H	8.70190000	-4.81720900	0.44392500
H	8.84247300	-4.68434000	-1.31963100
H	9.65737900	-5.99866600	-0.47119300
H	6.69767700	-7.71609100	-2.10704000
H	7.49068100	-6.31163600	-2.83900900
H	8.46852200	-7.60370300	-2.11295600
H	1.95278900	2.14717000	4.19834400
H	2.23079100	3.41080100	2.96547800
H	1.72692900	1.78817000	2.47999200
H	3.08010600	-0.15849200	-5.10764600
H	4.26402700	1.02004000	-4.56745900
H	4.26408200	-1.02005300	4.56747000
H	3.08009900	0.15841900	5.10764900

Cartesian coordinates of the optimized geometry for *cis,trans*-**10** at B3LYP-D3/6-31G* level of theory:

O	2.46733400	2.11625900	3.31572800
O	3.75685500	5.26377700	-0.61400100
N	0.68456800	-4.60798500	-1.34854700
N	0.75318900	-4.79127500	-0.10416600
N	5.15295600	-1.23661900	2.74123400
N	5.60949000	0.83379200	2.08279700
N	4.21031300	3.13164900	2.23382200
N	4.46539400	3.10438200	-0.47959500
C	-3.06710100	-3.62831100	-2.97958100
C	-2.67065300	-3.12781500	-1.73658900
C	-1.47360600	-3.53034900	-1.15624500
C	-0.63097400	-4.42417400	-1.83407800
C	-1.00906800	-4.92021700	-3.08971100
C	-2.23052400	-4.54161100	-3.63982200
C	2.00224000	-4.45437500	0.47261000
C	2.34716500	-5.00557000	1.71110400
C	3.40331800	-4.46607000	2.43972800
C	4.10227800	-3.34969200	1.96231500
C	3.76542400	-2.82396200	0.70911000
C	2.73651900	-3.37550600	-0.04039700
C	5.19136500	-2.70476600	2.80104400
C	6.03549600	-0.40878400	2.07371200
C	4.10997100	-0.45237300	3.20764000
C	4.41883400	0.83083400	2.79025900
C	3.60896800	2.06186100	2.84635200
C	3.46043100	4.15940900	1.52308500
C	3.91404700	4.23659500	0.04431900
C	5.04793500	3.05140800	-1.82401700
C	2.94956300	-1.00227000	3.96812400
C	3.51051800	5.54517900	2.20155500
C	4.95319600	6.04011200	2.36338300
C	2.77175800	5.49871800	3.54418000
C	6.59610500	3.15357300	-1.79504200
C	7.14669400	3.18223100	-3.22776600
C	7.06101000	4.38133000	-1.00267500
O	3.81457300	-2.66662300	-2.89714600
O	7.47474700	-3.56677400	0.45362000
N	-2.49470600	5.35584300	-1.75344900
N	-2.02146200	5.18377400	-2.89647200
N	3.58181300	1.64985700	-3.41408400
N	5.07852300	0.59407300	-2.15033100
N	5.96807100	-2.01598600	-2.44567800
N	6.80568300	-1.42763800	0.03703400
C	-1.25027200	3.20633200	1.67028000
C	-1.19825400	4.60703200	1.65085500
C	-1.59579200	5.31649000	0.52194500
C	-1.98475900	4.62215300	-0.62789900
C	-2.03300600	3.22481800	-0.62566200
C	-1.68771400	2.53007000	0.52856500
C	-0.80339900	4.44808800	-3.09895000
C	-0.76837900	3.43696800	-4.06400000
C	0.45142600	2.84303100	-4.38577200
C	1.64429300	3.26685800	-3.78229100
C	1.59303500	4.28762100	-2.82578700
C	0.38037900	4.86951300	-2.47794800
C	2.98525400	2.72608300	-4.23156900
C	4.59654300	1.77481600	-2.48543200
C	3.40173700	0.29574200	-3.64962500
C	4.34820500	-0.33356400	-2.87088300
C	4.65072900	-1.77600400	-2.75648700
C	6.41370200	-3.16648500	-1.67771400

C	6.94945000	-2.74392900	-0.29368800
C	7.25153600	-0.88279700	1.31939500
C	2.37950900	-0.27321900	-4.57375900
C	7.44918900	-4.03905700	-2.42325900
C	8.72738100	-3.25243600	-2.74487900
C	6.82213300	-4.63718100	-3.68722200
C	8.30482200	0.23633400	1.13266900
C	8.85124800	0.69720900	2.49143100
C	9.44348100	-0.24163900	0.22214700
O	-5.20924800	5.39305800	0.14550600
O	-3.71294300	4.26254800	3.99910600
O	0.15756900	-2.82056400	2.04681500
O	-2.63994900	-6.34555500	0.74407500
O	-5.94578100	-5.47178500	-1.83618000
O	-3.83029400	2.28072400	-3.30802600
N	-5.01000700	3.25894900	0.74356600
N	-4.10747300	2.17974500	3.13366200
N	-2.43521200	-0.74053900	3.47730800
N	-1.25332200	1.08290300	2.99549700
N	-1.93183100	-3.39386000	2.77810400
N	-3.85875400	-4.50077400	0.99378700
N	-5.83794700	-3.50261400	-0.67215300
N	-5.91314300	-0.36533000	-1.94653400
N	-4.85209300	-1.90291200	-3.14900900
N	-5.62101600	2.35719800	-1.87851200
C	-5.59521400	3.78653400	-1.66005400
C	-5.29250500	4.07744600	-0.21793000
C	-4.67903000	4.08082200	1.82553000
C	-4.80108800	5.38775100	1.45592300
C	-4.56973400	6.68763100	2.12726400
C	-4.13705000	3.54121700	3.09029800
C	-3.45978600	1.43386300	4.21109500
C	-4.46302600	0.63482100	5.07780900
C	-5.63356300	1.53185600	5.50227800
C	-3.75933000	0.04106000	6.30726900
C	-6.88898700	4.49231600	-2.14988000
C	-7.02772600	4.34038500	-3.66865300
C	-8.13527300	3.98029400	-1.41528600
C	-2.38828000	0.56733200	3.59983600
C	-1.29386400	-1.09326600	2.78340400
C	-0.55381400	0.02448000	2.44694700
C	0.66971800	0.17993400	1.60793100
C	-0.94606400	-2.49005100	2.49802800
C	-1.79866000	-4.79297400	2.43300200
C	-2.82413900	-5.16201800	1.40297900
C	-4.40106100	-5.28555900	-0.02920700
C	-3.64838800	-6.41225700	-0.18525100
C	-3.66170000	-7.55505800	-1.12791800
C	-5.47681800	-4.79558800	-0.91374800
C	-6.68847000	-2.72978400	-1.57700800
C	-7.94831100	-2.17179500	-0.87270100
C	-8.65790100	-3.28383400	-0.08840800
C	-8.90395000	-1.53464800	-1.89271900
C	-1.83392700	-5.72931600	3.67185600
C	-3.15890900	-5.61940900	4.43760000
C	-0.63577400	-5.43398800	4.58195700
C	-5.85118700	-1.64870400	-2.22371600
C	-4.93041700	0.24333400	-2.70605400
C	-4.25136100	-0.69515900	-3.45713800
C	-3.11255000	-0.57105100	-4.41203400
C	-4.72455500	1.70583500	-2.67097200
C	-0.84622900	2.48504400	2.94220200
C	-4.37741600	-3.20191300	-3.62289000
H	5.17558700	2.97600600	1.96731000
H	4.59561700	2.28467200	0.10230500
H	-3.29304600	-2.40850300	-1.21664000

H	-1.14047400	-3.11722100	-0.21058000
H	-0.33592800	-5.58306800	-3.62502900
H	-2.52497300	-4.93398500	-4.61069800
H	1.75495900	-5.82229900	2.11211100
H	3.65207300	-4.88208900	3.41321100
H	4.27909600	-1.94514900	0.33561300
H	2.45595900	-2.94802100	-0.99508100
H	6.17373400	-3.02905800	2.44636400
H	5.09875600	-3.00986000	3.84826900
H	2.41382400	3.82667500	1.50970400
H	4.65780100	3.92360800	-2.35302400
H	2.31665500	-1.64043200	3.33867300
H	3.28524700	-1.60554100	4.82101200
H	2.34542400	-0.17302100	4.33811400
H	2.98561500	6.22894800	1.52512700
H	4.97023800	7.03537100	2.82090500
H	5.45919200	6.10945500	1.39405100
H	5.52622000	5.36690500	3.01302200
H	2.79316500	6.48113700	4.02953300
H	1.72384600	5.20466200	3.41177400
H	3.23178000	4.77037600	4.22073200
H	6.96939600	2.24773100	-1.29866000
H	6.85779700	2.28830600	-3.79064100
H	6.77835000	4.06365800	-3.76950000
H	8.24074000	3.23416000	-3.21937700
H	6.64104100	5.30336700	-1.42093700
H	8.15356600	4.45858100	-1.02910300
H	6.75692900	4.32500800	0.04693600
H	6.57827600	-1.21029000	-2.47683100
H	6.27298700	-0.80024300	-0.55931100
H	-0.89453100	5.14525700	2.54512300
H	-1.59822600	6.40262600	0.51755000
H	-2.37988400	2.70077400	-1.50937600
H	-1.78426600	1.44961400	0.54051800
H	-1.69644500	3.12090200	-4.52881000
H	0.47763800	2.06452900	-5.14437100
H	2.49791000	4.65672900	-2.35347700
H	0.35475500	5.66893100	-1.74541200
H	3.71836000	3.53540400	-4.26262600
H	2.90997100	2.33534300	-5.24989500
H	5.52032200	-3.77184000	-1.48938400
H	7.72738000	-1.71514700	1.84567500
H	1.36776600	0.01659600	-4.26787900
H	2.52253200	0.05903700	-5.61053300
H	2.44952700	-1.36203500	-4.53762600
H	7.70790600	-4.84606600	-1.72840700
H	9.46004200	-3.89552800	-3.24462100
H	9.19640400	-2.85732800	-1.83633900
H	8.51565600	-2.41391400	-3.42123800
H	7.53289500	-5.29915400	-4.19502700
H	5.92080700	-5.21373500	-3.45174500
H	6.53231200	-3.84580500	-4.38789200
H	7.80099100	1.08807100	0.65658800
H	8.05776900	1.08954700	3.13288700
H	9.34159300	-0.13429500	3.01460100
H	9.59789600	1.48718700	2.35399000
H	9.93876900	-1.12444200	0.64568800
H	10.19663800	0.54612500	0.11019700
H	9.08268800	-0.50979100	-0.77526600
H	-4.40710500	1.68623300	2.30063600
H	-2.83412700	-3.02284900	3.04838300
H	-5.35596500	-3.02312300	0.07979400
H	-6.23326200	1.77413500	-1.32043000
H	-4.75573300	4.17374600	-2.24836100
H	-3.84536400	7.28596500	1.56245700
H	-4.18105200	6.49258100	3.12792100

H	-5.49725600	7.26841200	2.20123500
H	-3.00632000	2.19882000	4.85136200
H	-4.84691900	-0.19224100	4.46630600
H	-5.27360900	2.40689500	6.05730100
H	-6.32156200	0.97703000	6.15011700
H	-6.19755900	1.89654100	4.63884600
H	-2.95461500	-0.63963300	6.01907900
H	-3.33600000	0.83791000	6.93332500
H	-4.47471500	-0.51655000	6.92245400
H	-6.75783000	5.55621300	-1.91314900
H	-7.14317100	3.28543600	-3.94213800
H	-7.90758000	4.88301000	-4.03225400
H	-6.14506800	4.72730100	-4.18985200
H	-8.31755700	2.92192600	-1.63974200
H	-9.02364100	4.53876500	-1.72976100
H	-8.03900800	4.08618800	-0.32818600
H	0.97743400	-0.80176300	1.24865200
H	1.48252400	0.62780300	2.17976000
H	0.47568300	0.83617100	0.75077200
H	-0.82302200	-4.89506400	1.94506500
H	-4.51981900	-7.44479900	-1.79227900
H	-2.74069600	-7.56400800	-1.72333900
H	-3.72905800	-8.51059700	-0.59452300
H	-7.01736400	-3.44682800	-2.33666300
H	-7.62027700	-1.39155000	-0.17327900
H	-8.95394700	-4.10343800	-0.75532400
H	-9.56352800	-2.89237600	0.38810400
H	-8.01699400	-3.70538300	0.69121600
H	-9.23433600	-2.27794900	-2.63051500
H	-9.79775800	-1.15238400	-1.38701700
H	-8.43218300	-0.70413200	-2.42330900
H	-1.73883300	-6.75219800	3.28137200
H	-4.02088000	-5.82280300	3.79170500
H	-3.28150100	-4.61462300	4.86006800
H	-3.18414800	-6.33152400	5.26970400
H	0.31116400	-5.51447300	4.03682100
H	-0.70044700	-4.41579200	4.98119600
H	-0.60974700	-6.13088000	5.42724500
H	-3.39308300	-0.90655500	-5.41923900
H	-2.81503700	0.47666000	-4.46060300
H	-2.25795600	-1.17731200	-4.08856400
H	0.24022100	2.51988400	3.07196500
H	-1.29102000	2.99958900	3.79851600
H	-5.15083700	-3.94806500	-3.42076300
H	-4.26059200	-3.14569400	-4.71048200

Cartesian coordinates of the optimized geometry for *cis*,*cis*-**10** at B3LYP-D3/6-31G* level of theory:

O	2.88054400	-3.81293100	-1.54490100
O	5.47418600	-3.78979000	2.81927200
N	-0.16599100	4.93247800	0.63276900
N	0.47483200	4.94018200	-0.43649800
N	4.02484700	0.05671100	-3.03880100
N	5.35804900	-1.19728700	-1.78640100
N	5.04145900	-3.72677600	-0.77889300
N	5.67130000	-2.10322400	1.30178100
C	-1.73521200	1.93394300	3.16550000
C	-1.82392100	1.75579300	1.78090500
C	-1.26491200	2.68157900	0.89710900
C	-0.59899600	3.80833700	1.39880100
C	-0.53070800	4.00210600	2.78855300
C	-1.07176200	3.06600900	3.65766800
C	1.08684100	3.84190700	-1.12388600
C	1.47428700	4.21663300	-2.42145200

C	2.21304300	3.36165000	-3.22537500
C	2.60744600	2.10675500	-2.74705000
C	2.23578800	1.73080400	-1.45160000
C	1.47811200	2.58077300	-0.64393200
C	3.46075200	1.24705800	-3.66458600
C	5.22469600	-0.02061900	-2.36105800
C	3.36946600	-1.15193000	-2.88060700
C	4.21812000	-1.91936700	-2.10767500
C	3.96821600	-3.24065000	-1.49618700
C	4.86177100	-4.32255500	0.54163000
C	5.37039200	-3.38608700	1.66195300
C	6.19576000	-1.12787200	2.25478100
C	2.02693100	-1.44462100	-3.45403800
C	5.48011600	-5.73061800	0.66710600
C	6.99092800	-5.70927100	0.40154200
C	4.75010100	-6.70222700	-0.26701500
C	7.62782900	-0.65440200	1.90429200
C	8.21518300	0.16934300	3.05952000
C	8.52975900	-1.84900100	1.57016600
O	2.88106300	3.81334400	1.54476500
O	5.47395700	3.78997100	-2.81988000
N	-0.16637500	-4.93251200	-0.63199900
N	0.47466400	-4.94013100	0.43714100
N	4.02526000	-0.05644000	3.03832500
N	5.35840400	1.19749500	1.78579800
N	5.04183100	3.72703200	0.77836300
N	5.67126200	2.10340200	-1.30241500
C	-1.73581400	-1.93415300	-3.16478400
C	-1.07229600	-3.06618100	-3.65695600
C	-0.53116700	-4.00223700	-2.78784800
C	-0.59941900	-3.80843100	-1.39809800
C	-1.26540500	-2.68172300	-0.89640500
C	-1.82452800	-1.75599900	-1.78019600
C	1.08686500	-3.84180500	1.12428300
C	1.47477100	-4.21651700	2.42171600
C	2.21370000	-3.36147700	3.22541700
C	2.60778700	-2.10651900	2.74699900
C	2.23565700	-1.73057200	1.45168300
C	1.47782700	-2.58061000	0.64423200
C	3.46124800	-1.24672500	3.66429900
C	5.22504600	0.02083000	2.36046300
C	3.36994800	1.15225400	2.88024900
C	4.21856300	1.91964300	2.10722800
C	3.96867300	3.24096400	1.49582600
C	4.86192800	4.32277700	-0.54215100
C	5.37036400	3.38628500	-1.66253700
C	6.19549800	1.12802300	-2.25551400
C	2.02746300	1.44501500	3.45376100
C	5.48024000	5.73084100	-0.66775900
C	6.99108100	5.70952300	-0.40236200
C	4.75031500	6.70247800	0.26640300
C	7.62758800	0.65445200	-1.90525200
C	8.21469900	-0.16932900	-3.06057900
C	8.52965300	1.84898600	-1.57126000
O	-3.67251900	-5.93880600	-1.41959500
O	-4.76786300	-3.16510200	-4.51003000
O	-1.76801600	4.05075800	-2.19085400
O	-3.67252700	5.93883500	1.41920800
O	-4.76678500	3.16504200	4.50998400
O	-1.76739300	-4.05139100	2.19275700
N	-4.49825600	-3.92499300	-0.95852600
N	-5.03928100	-1.82285000	-2.67322300
N	-4.41114000	1.61084200	-2.67159000
N	-2.96558700	0.20349600	-3.60325700
N	-4.01578900	4.23033100	-1.79809300
N	-4.49817700	3.92491900	0.95841300

N	-5.03846000	1.82272900	2.67325100
N	-4.41034900	-1.61095000	2.67188300
N	-2.96498400	-0.20372200	3.60401900
N	-4.01482400	-4.23014100	1.79769500
C	-3.87801700	-5.41640800	0.97362600
C	-4.04125800	-5.03187000	-0.46786300
C	-4.42402600	-4.09336000	-2.34279100
C	-3.91109500	-5.32532200	-2.62522600
C	-3.54446200	-6.04391300	-3.86712400
C	-4.77216800	-3.01265800	-3.28257600
C	-5.37298400	-0.60598400	-3.40688600
C	-6.77817200	-0.06161300	-3.04474800
C	-7.80611900	-1.20163500	-3.01902400
C	-7.21169400	1.02851800	-4.03724300
C	-4.84906600	-6.55289800	1.39037600
C	-4.48816600	-7.06848600	2.78799000
C	-6.31740100	-6.11155400	1.31531200
C	-4.27812500	0.42040400	-3.21396100
C	-3.15767400	2.19395000	-2.71192800
C	-2.24020300	1.33205900	-3.27991800
C	-0.77689100	1.46056900	-3.52023400
C	-2.89989300	3.55679400	-2.21832400
C	-3.87929600	5.41659900	-0.97397000
C	-4.04155800	5.03188400	0.46759800
C	-4.42338400	4.09324800	2.34265200
C	-3.91050600	5.32526900	2.62491900
C	-3.54345400	6.04386100	3.86669300
C	-4.77129000	3.01255700	3.28253600
C	-5.37227600	0.60591800	3.40694200
C	-6.77741600	0.06155900	3.04459500
C	-7.80531200	1.20161800	3.01851800
C	-7.21118900	-1.02843200	4.03713400
C	-4.85134200	6.55244900	-1.39012400
C	-6.31935400	6.11011000	-1.31444300
C	-4.49146600	7.06853700	-2.78781900
C	-4.27740800	-0.42051500	3.21428400
C	-3.15696700	-2.19420600	2.71273000
C	-2.23961100	-1.33239000	3.28102000
C	-0.77641700	-1.46106300	3.52196600
C	-2.89914200	-3.55707800	2.21919700
C	-2.39200500	-1.00660000	-4.17457400
C	-2.39144500	1.00641600	4.17529600
H	5.90737000	-3.22463500	-0.93150800
H	5.53258400	-1.78045200	0.34825000
H	-2.35344900	0.89512800	1.38239100
H	-1.35921400	2.54985300	-0.17455400
H	-0.04470100	4.89957300	3.15847600
H	-1.01098900	3.22905100	4.73147400
H	1.18996400	5.20566900	-2.76334500
H	2.52435900	3.68452100	-4.21524200
H	2.55984500	0.77294300	-1.05308800
H	1.24724500	2.28140000	0.36714600
H	4.28307300	1.86040900	-4.04281400
H	2.87480500	0.92071200	-4.53039700
H	3.78130600	-4.41330800	0.69548900
H	6.24369300	-1.65834900	3.21101900
H	1.26668000	-0.75677800	-3.06792100
H	2.02303500	-1.36079900	-4.54861700
H	1.73838000	-2.45743700	-3.17640400
H	5.31550500	-6.03445600	1.70706200
H	7.41980300	-6.70979500	0.52522700
H	7.51026600	-5.03582700	1.09376300
H	7.20342400	-5.38551000	-0.62540300
H	5.15009100	-7.71706400	-0.15934200
H	3.67564500	-6.72903200	-0.05263700
H	4.86862700	-6.39564600	-1.31241000

H	7.55944300	-0.00394100	1.02459900
H	7.59734500	1.04263200	3.28677400
H	8.30094800	-0.44121500	3.96790900
H	9.21939800	0.52415700	2.80275400
H	8.56093500	-2.56148400	2.40388800
H	9.55300900	-1.50892700	1.37634300
H	8.18005200	-2.38851900	0.68496600
H	5.90773300	3.22483000	0.93082400
H	5.53264600	1.78062100	-0.34887300
H	-1.01150900	-3.22921100	-4.73076300
H	-0.04510000	-4.89967100	-3.15776900
H	-1.35961000	-2.54992400	0.17526100
H	-2.35410900	-0.89536900	-1.38167400
H	1.19064300	-5.20558400	2.76367900
H	2.52539500	-3.68434800	4.21516400
H	2.55946600	-0.77265700	1.05309600
H	1.24661500	-2.28123100	-0.36676400
H	4.28362400	-1.86004200	4.04245900
H	2.87543900	-0.92029700	4.53017200
H	3.78143500	4.41351900	-0.69584000
H	6.24331600	1.65850800	-3.21175300
H	1.73917300	2.45802900	3.17657800
H	1.26705000	0.75756200	3.06726100
H	2.02346800	1.36066900	4.54829700
H	5.31550600	6.03463300	-1.70770900
H	7.41993100	6.71004800	-0.52612700
H	7.51035100	5.03606100	-1.09461800
H	7.20369200	5.38579500	0.62457000
H	5.15027500	7.71731700	0.15863800
H	3.67583300	6.72925100	0.05215200
H	4.86896600	6.39594500	1.31179700
H	7.55929600	0.00398900	-1.02555300
H	7.59676800	-1.04257900	-3.28773000
H	8.30035200	0.44122200	-3.96898300
H	9.21893400	-0.52420800	-2.80398100
H	8.56075800	2.56146600	-2.40498800
H	9.55290600	1.50883800	-1.37758700
H	8.18011200	2.38853100	-0.68601100
H	-4.98766900	-1.80174100	-1.66112000
H	-4.84509900	3.65875400	-1.67906000
H	-4.98707200	1.80163200	1.66113600
H	-4.84393400	-3.65837800	1.67818400
H	-2.84906200	-5.76684600	1.10606100
H	-2.46890600	-6.25667300	-3.87883200
H	-3.80131100	-5.41254200	-4.71903700
H	-4.07770300	-6.99865400	-3.94570700
H	-5.39268700	-0.91442200	-4.45885100
H	-6.71485000	0.38762300	-2.04448900
H	-7.84009300	-1.71722200	-3.98703600
H	-8.80580800	-0.80302400	-2.81378700
H	-7.57437700	-1.94811900	-2.25375900
H	-6.51340200	1.86810700	-4.04323400
H	-7.27745400	0.61932800	-5.05410900
H	-8.20368300	1.41022400	-3.77038900
H	-4.69222400	-7.36263500	0.66452500
H	-4.59282100	-6.26937400	3.53009200
H	-5.14812300	-7.89360400	3.07851500
H	-3.45371300	-7.42851000	2.82500800
H	-6.52981800	-5.33126700	2.05643600
H	-6.98260400	-6.95526100	1.52853100
H	-6.57606000	-5.71834900	0.32540000
H	-0.47359800	2.48727700	-3.32609300
H	-0.50512100	1.19384200	-4.54923500
H	-0.20972400	0.80804900	-2.84688700
H	-2.85064700	5.76771900	-1.10689900
H	-3.80006500	5.41251000	4.71869400

H	-2.46788500	6.25656500	3.87806400
H	-4.07663000	6.99862400	3.94542400
H	-5.39214000	0.91442100	4.45888400
H	-6.71391400	-0.38780400	2.04440400
H	-7.83947900	1.71731600	3.98646400
H	-8.80497300	0.80302200	2.81311100
H	-7.57338100	1.94800900	2.25321700
H	-7.27710100	-0.61912500	5.05394300
H	-8.20315800	-1.41009700	3.77014700
H	-6.51296100	-1.86807100	4.04333700
H	-4.69473700	7.36217900	-0.66421400
H	-6.57721100	5.71629800	-0.32456600
H	-6.53171400	5.33003200	-2.05580500
H	-6.98520900	6.95348700	-1.52692400
H	-3.45732700	7.42942900	-2.82522200
H	-4.59578100	6.26945300	-3.52999700
H	-5.15221800	7.89316300	-3.07793500
H	-0.50514900	-1.19484000	4.55123300
H	-0.47310000	-2.48770800	3.32751800
H	-0.20889100	-0.80825000	2.84920900
H	-1.65888700	-0.70400400	-4.92967900
H	-3.17946100	-1.54876000	-4.70525700
H	-3.17895000	1.54862200	4.70586500
H	-1.65838000	0.70387100	4.93046800

Cartesian coordinates of the optimized geometry for *trans*-azobenzene at B3LYP-D3/6-31G* level of theory:

C	4.01334500	1.09546600	-0.00001500
C	2.63243500	1.28634200	0.00002700
C	1.76797300	0.18354700	0.00006200
C	2.29489500	-1.12029200	-0.00000900
C	3.67260400	-1.30352300	-0.00007000
C	4.53550500	-0.19934000	-0.00005700
H	4.67967800	1.95357000	-0.00001900
H	2.19665000	2.28094000	0.00006100
H	1.61042400	-1.96116200	-0.00000900
H	4.08261200	-2.31010500	-0.00011400
H	5.61141000	-0.35233000	-0.00009900
N	0.38524300	0.49871300	0.00010200
N	-0.38523300	-0.49867300	0.00011500
C	-1.76797000	-0.18352700	0.00003200
C	-2.63241400	-1.28633900	0.00003800
C	-2.29491600	1.12030100	-0.00001400
C	-4.01332500	-1.09548800	-0.00003800
H	-2.19660600	-2.28092600	0.00008200
C	-3.67263100	1.30350700	-0.00006400
H	-1.61046400	1.96118600	0.00000200
C	-4.53551000	0.19930900	-0.00008300
H	-4.67964600	-1.95360100	-0.00004800
H	-4.08265400	2.31008300	-0.00009600
H	-5.61141800	0.35227500	-0.00013000

Cartesian coordinates of the optimized geometry for *cis*-azobenzene at B3LYP-D3/6-31G* level of theory:

C	-3.39435500	-0.39166500	0.67714600
C	-2.52679100	0.69518800	0.76869500
C	-1.41753400	0.77613200	-0.08195400
C	-1.21975300	-0.19675100	-1.07290500
C	-2.11320100	-1.25984600	-1.18124200
C	-3.19213100	-1.37046300	-0.29936400
H	-4.23980300	-0.46453400	1.35576400
H	-2.68688500	1.48389100	1.49796000

H	-0.37662500	-0.11247600	-1.75025500
H	-1.96329600	-2.00822600	-1.95469800
H	-3.88000000	-2.20712200	-0.38325500
N	-0.62486200	1.97044900	0.00588700
N	0.62487300	1.97044800	-0.00587300
C	1.41754100	0.77612800	0.08195800
C	2.52681800	0.69520000	-0.76866600
C	1.21973000	-0.19678000	1.07287800
C	3.39437500	-0.39165900	-0.67712400
H	2.68693400	1.48392100	-1.49790800
C	2.11317000	-1.25988200	1.18121000
H	0.37658200	-0.11251900	1.75020600
C	3.19212200	-1.37048000	0.29935700
H	4.23984000	-0.46451500	-1.35572200
H	1.96324100	-2.00828100	1.95464100
H	3.87998500	-2.20714600	0.38324300

4. ^{1}H NMR and ^{13}C NMR spectra of the chiral container 10

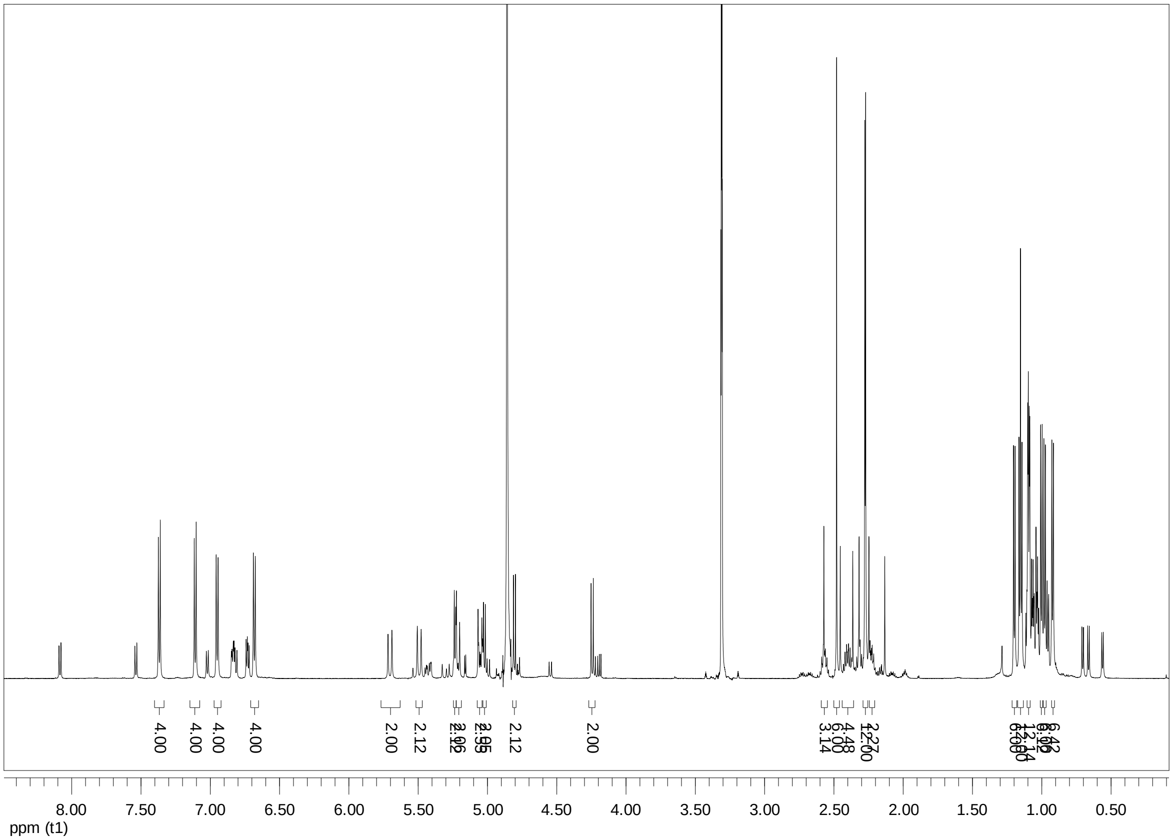

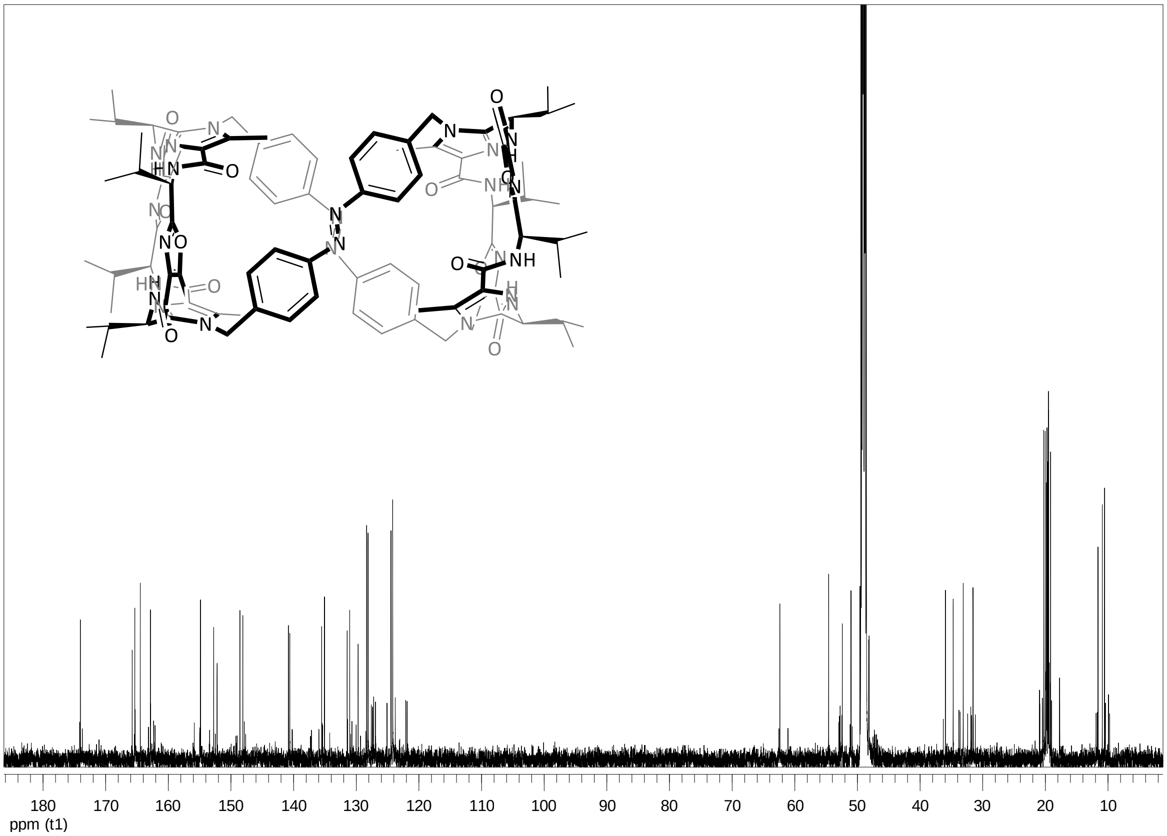

Supplement: File 1 — Molecular structures, HPLC spectra of the foldable container, cartesian coordinates and absolute energies for all calculated compounds, as well as the NMR spectra of the new chiral container. [file Beilstein_J_Org_Chem-15-1534-s001.pdf]
